# Firefighting and Cancer: A Meta-analysis of Cohort Studies in the Context of Cancer Hazard Identification

**DOI:** 10.1016/j.shaw.2023.02.003

**Published:** 2023-03-07

**Authors:** Nathan L. DeBono, Robert D. Daniels, Laura E. Beane Freeman, Judith M. Graber, Johnni Hansen, Lauren R. Teras, Tim Driscoll, Kristina Kjaerheim, Paul A. Demers, Deborah C. Glass, David Kriebel, Tracy L. Kirkham, Roland Wedekind, Adalberto M. Filho, Leslie Stayner, Mary K. Schubauer-Berigan

**Affiliations:** 1 Evidence Synthesis and Classification Branch, International Agency for Research on Cancer, Lyon, France; 2 National Institute for Occupational Safety and Health, Centers for Disease Control and Prevention, Cincinnati, USA; 3 Occupational and Environmental Epidemiology Branch, Division of Cancer Epidemiology and Genetics, National Cancer Institute, Rockville, USA; 4 Department of Biostatistics and Epidemiology, Rutgers School of Public Health, Piscataway, USA; 5 Danish Cancer Society Research Centre, Copenhagen, Denmark; 6 American Cancer Society, Atlanta, USA; 7 Sydney School of Public Health, Faculty of Medicine and Health, University of Sydney, Sydney, Australia; 8 Department of Research, Cancer Registry of Norway, Oslo, Norway; 9 Occupational Cancer Research Centre, Ontario Health, Toronto, Canada; 10 School of Epidemiology and Preventative Medicine, Monash University, Melbourne, Australia; 11 Lowell Center for Sustainable Production, University of Massachusetts Lowell, Lowell, USA; 12 University of Illinois at Chicago, School of Public Health, Division of Epidemiology and Biostatistics

**Keywords:** Cancer, Firefighter, Hazard

## Abstract

**Objective:**

We performed a meta-analysis of epidemiological results for the association between occupational exposure as a firefighter and cancer as part of the broader evidence synthesis work of the *IARCMonographs* program.

**Methods:**

A systematic literature search was conducted to identify cohort studies of firefighters followed for cancer incidence and mortality. Studies were evaluated for the influence of key biases on results. Random-effects meta-analysis models were used to estimate the association between ever-employment and duration of employment as a firefighter and risk of 12 selected cancers. The impact of bias was explored in sensitivity analyses.

**Results:**

Among the 16 included cancer incidence studies, the estimated meta-rate ratio, 95% confidence interval (CI), and heterogeneity statistic (I^2^) for ever-employment as a career firefighter compared mostly to general populations were 1.58 (1.14–2.20, 8%) for mesothelioma, 1.16 (1.08–1.26, 0%) for bladder cancer, 1.21 (1.12–1.32, 81%) for prostate cancer, 1.37 (1.03–1.82, 56%) for testicular cancer, 1.19 (1.07–1.32, 37%) for colon cancer, 1.36 (1.15–1.62, 83%) for melanoma, 1.12 (1.01–1.25, 0%) for non-Hodgkin lymphoma, 1.28 (1.02–1.61, 40%) for thyroid cancer, and 1.09 (0.92–1.29, 55%) for kidney cancer. Ever-employment as a firefighter was not positively associated with lung, nervous system, or stomach cancer. Results for mesothelioma and bladder cancer exhibited low heterogeneity and were largely robust across sensitivity analyses.

**Conclusions:**

There is epidemiological evidence to support a causal relationship between occupational exposure as a firefighter and certain cancers. Challenges persist in the body of evidence related to the quality of exposure assessment, confounding, and medical surveillance bias.

## 1 Introduction

Firefighting is a complex activity that involves potential exposure to a variety of carcinogenic hazards resulting from fires and other emergency events. Firefighters can be exposed to many carcinogenic hazards, including combustion products (e.g., polycyclic aromatic hydrocarbons [PAHs], and particulates), asbestos, chemicals in firefighting foams (e.g., perfluorinated and polyfluorinated substances [PFAS]), flame retardants, diesel exhaust, ultraviolet radiation, and night shift work. Biological uptake of fire effluents and other chemicals can occur through dermal absorption, inhalation, and ingestion. The tasks, responsibilities, equipment, and employment status (e.g., full-time, volunteer) of firefighters have also evolved significantly over time and across countries. As climate change becomes increasingly severe, wildland fires are expected to become more common and will encroach more frequently on urban areas. Estimates drawn from 56 countries suggest that more than 15 million firefighters, both full-time and part-time, worked during 2010–2019, making the primary prevention of cancer among firefighters a critical issue in occupational health [1].

Despite firefighters' potential for exposure to many known and suspected carcinogens at work, there has been notable inconsistency in epidemiological research regarding the presence and magnitude of cancer risk attributable to the occupation and the specific cancer types of greatest concern. In 2007, the *IARC* *Monographs* program classified occupational exposure as a firefighter as “possibly carcinogenic to humans” (Group 2B) based on “limited” evidence in humans for cancers of the prostate, testis, and non-Hodgkin lymphoma (NHL) [2]. Since this evaluation, several cohort studies have been published that provide results based on a range of exposure definitions, study population characteristics, comparison groups, and cancer outcomes. Reconciling these differences as well as the influence of known biases, such as confounding, medical surveillance bias, and healthy worker hire and survivor bias, is a significant challenge for evidence synthesis efforts on this topic.

We sought to meta-analyze results from epidemiological studies of the association between occupational exposure as a firefighter and the occurrence of cancer. Thirteen cancer sites were chosen *a priori* for meta-analysis and were selected based on evidence from previous reviews on the topic. While several previous meta-analyses have been conducted for these cancer sites [[3], [4], [5], [6], [7], [8], [9]], new studies have since become available. This effort was undertaken as part of the broader systematic review and evidence synthesis work in the human cancer section of volume 132 of the *IARC* *Monographs* program evaluation of the carcinogenicity of occupational exposure as a firefighter [10]. The overarching objective of the *Monographs* evaluation was to determine whether evidence of a carcinogenic hazard exists among individuals working or volunteering in the firefighting occupation by reviewing both mechanistic and epidemiological data. The present meta-analysis was used by the *Monographs* volume 132 Working Group in June 2022 to support the achievement of this objective using the most recent available evidence.

## 2 Methods

A detailed description of the methods is provided in the supplemental material. Briefly, a systematic search was conducted of three literature databases to identify epidemiological studies of the association between occupational exposure as a firefighter and the occurrence of cancer incidence or mortality in humans published until June 13, 2022. Occupational exposure as a firefighter was defined as any exposure to the occupation regardless of employment type (e.g., career or part-time) or activities performed (e.g., wildland or structural firefighting). The exact search terms, results, and a flowchart illustrating the number of included and excluded studies are available in Suppl. Table A and Suppl. Fig. 1. After exclusion criteria were applied, 63 studies received a detailed full-text review. Only one population-based case-control study met inclusion criteria, but it was excluded from meta-analyses with cohort studies to reduce the heterogeneity of estimated effects due to marked differences in study design [12]. Results from this study were instead synthesized qualitatively with meta-analysis results. The protocol for this meta-analysis was registered with the international database of prospectively registered systematic reviews (PROSPERO) with a registration ID of CRD42021258545.

A bias assessment tool was developed to evaluate the potential influence of six biases determined through the Working Group's judgment to be most relevant to epidemiological studies of occupational exposure as a firefighter and cancer. The chosen bias domains were misclassification of exposure, misclassification of outcome, healthy worker hire and survivor bias, confounding by lifestyle factors (e.g., tobacco or alcohol consumption, sun exposure) or occupational exposures outside of firefighting, medical surveillance bias, and selection bias. Studies identified as having a “major” level of concern for one or more bias domains were excluded in sensitivity analyses (described below) to determine the impact of results from these studies on the meta-effect estimates.

The objective of the analysis was to meta-analyze the association between ever-employment and duration of employment as a firefighter and cancer incidence and mortality. To reduce the heterogeneity of included study populations and exposure to the occupation, results based exclusively on females or part-time/volunteer firefighters were excluded from analyses. Results for female firefighters were too few for stratified meta-analysis. Results from internal comparison analyses according to metrics of firefighting exposure were also excluded as few studies conducted such analyses, and the type of reported exposure metrics varied [[13], [14], [15], [16], [17]].

There were 13 cancer sites chosen for analysis identified *a priori* from the studies in the systematic literature review and the results from previous meta-analyses [[3], [4], [5]]. The specific studies included in the meta-analyses for each cancer site are listed in Suppl. Table B and C. The meta-effect estimates, referred to henceforth as meta-rate ratios (mRR), were estimated with inverse-variance weighted random-effects models and the natural logarithm of the reported study effect estimates. Estimates of within-study variance were specified in the models using the 95% confidence interval (CI) bounds reported in each study rather than a calculation of the standard error. Interval bounds for estimates from individual studies shown in forest plots may differ slightly from reported values, particularly for estimates based on few cases, but this had a negligible impact on results. The between-study variance (τ^2^) was estimated using restricted maximum-likelihood (REML) methods. Residual heterogeneity was described by the I^2^ statistic and Q test *p*-value [18,19]. The Hartung-Knapp-Sidik-Jonkman (HKSJ) method was used to calculate 95% CIs unless the interval was narrower than that using standard random-effects methods. Funnel plots were examined for evidence of reporting bias and are shown in Suppl. Fig. 2. All analyses were conducted using the “meta” package in R Statistical Software version 4.1.2.

The main analysis consisted of results for the association between ever-employment as a firefighter and cancer using any population as the referent. Results using general population referents were preferred when other populations were also available in a given study. All studies in the main analysis were cohort studies following firefighters for cancer incidence or mortality over time. A secondary analysis consisted of results for the association between duration of employment as a firefighter and cancer using a three-level mixed-effect model in both categorical meta-analysis and meta-regression.

Triangulation methods were used in sensitivity analyses to elucidate sources of bias and heterogeneity in mRR estimates. The impact of the use of alternative referent populations (e.g., police, military, other workers, or firefighters) was explored by preferring these results from a given study when available and through restriction. Such restriction was also applied to include only studies with older age (≥55 years at end of follow-up) or longer periods (>20 years) of follow-up to evaluate the influence of excluding studies that primarily observed cancer occurrence during younger, lower-risk age windows. Studies assessed as having a “major” level of concern for any of the six bias domains in the bias assessment exercise were also excluded separately to assess the potential impact of these sources of bias on results in the main analysis.

## 3 Results

After all exclusions during data analysis were applied, a total of 35 cohort studies were included in the meta-analysis (Table 1) [13,14,16,17,[20], [21], [22], [23], [24], [25], [26], [27], [28], [29], [30], [31], [32], [33], [34], [35], [36], [37], [38], [39], [40], [41], [42], [43], [44], [45], [46], [47]]. Study populations were from the USA, Canada, Northern and Western Europe, Australia, New Zealand, and the Republic of Korea. The most common concerns identified from the bias assessment exercise were medical surveillance bias [23,26,27,[32], [33], [34], [35]] (seven studies) and healthy worker bias [14,23,25,29,44] (five studies). No studies reported risk specific to exposure as a wildland firefighter. Studies of cancer incidence were fewer in number than those of mortality and tended to have more recent calendar periods of follow-up.

**Table 1:** Descriptive characteristics and bias assessment results of all studies included in meta-analysis∗

Reference	Location	Outcome	Mean age ≥55 years at follow-up end or mean >20 years follow-up	“Major” concern for surveillance bias	“Major” concern for healthy worker bias	“Major” concern for exposure misclassification
Marjerrison et al. (2022b)	Norway	Both	Yes	—	—	—
Sritharan et al. (2022)	Canada	Incidence	Yes	—	—	—
Marjerrison et al. (2022a)	Norway	Incidence	Yes	—	—	—
Webber et al. (2021)	USA	Incidence	—	Yes	Yes	—
Zhao et al. (2020)	Spain	Mortality	—	—	—	—
Pinkerton et al. (2020)†	USA	Mortality	Yes	—	—	—
Bigert et al. (2020)	Sweden	Incidence	Yes	—	Yes	Yes
Petersen et al. (2018a)‡	Denmark	Incidence	Yes	Yes	—	—
Petersen et al. (2018b)	Denmark	Mortality	Yes	Yes	—	—
Harris et al. (2018)	Canada	Incidence	—	—	—	—
Glass et al. (2016)	Australia	Both	—	—	Yes	—
Ahn and Jeong (2015)	Republic of Korea	Mortality	—	—	Yes	—
Amadeo et al. (2015)	France	Mortality	Yes	—	—	—
Pukkala et al. (2014)	Nordic	Incidence	Yes	—	—	—
Daniels et al. (2014)†	USA	Both	Yes	Yes	—	—
Ahn et al. (2012)	Republic of Korea	Incidence	—	Yes	—	—
Zeig-Owens et al. (2011)	USA	Incidence	—	Yes	Yes	—
Ma et al. (2006)	USA	Incidence	—	Yes	—	—
Ma et al. (2005)	USA	Mortality	—	—	—	—
Bates et al. (2001)	New Zealand	Both	—	—	—	—
Baris et al. (2001)	USA	Mortality	—	Yes		
Deschamps et al. (1995)	France	Mortality	—	—	—	—
Demers et al. (1994)	USA	Incidence	—	—	—	—
Tornling et al. (1994)	Sweden	Mortality	Yes	—	—	—
Aronson et al. (1994)	Canada	Mortality	Yes	—	—	—
Guidotti (1993)	Canada	Mortality	—	—	—	—
Giles et al. (1993)	Australia	Incidence	—	—	—	—
Demers et al. (1992)	USA	Mortality	Yes	—	—	—
Beaumont et al. (1991)	USA	Mortality	—	Yes		
Hansen (1990)	Denmark	Mortality	—	—	—	—
Heyer et al. (1990)	USA	Mortality	—	—		
Vena and Fiedler (1987)	USA	Mortality	—	—	Yes	—
Eliopulos et al. (1984)	Australia	Mortality	—	—	—	—
Musk et al. (1978)	USA	Mortality	—	—	—	—
Mastromatteo (1959)	Canada	Mortality	—	—	—	—

∗ The lack of a given characteristic in each study is denoted by the symbol “—“.

† Study population includes a small number of females.

‡ Study population include part-time/volunteer firefighters for some cancer sites.

### 3.1 Main analysis

**Fig. 1:**
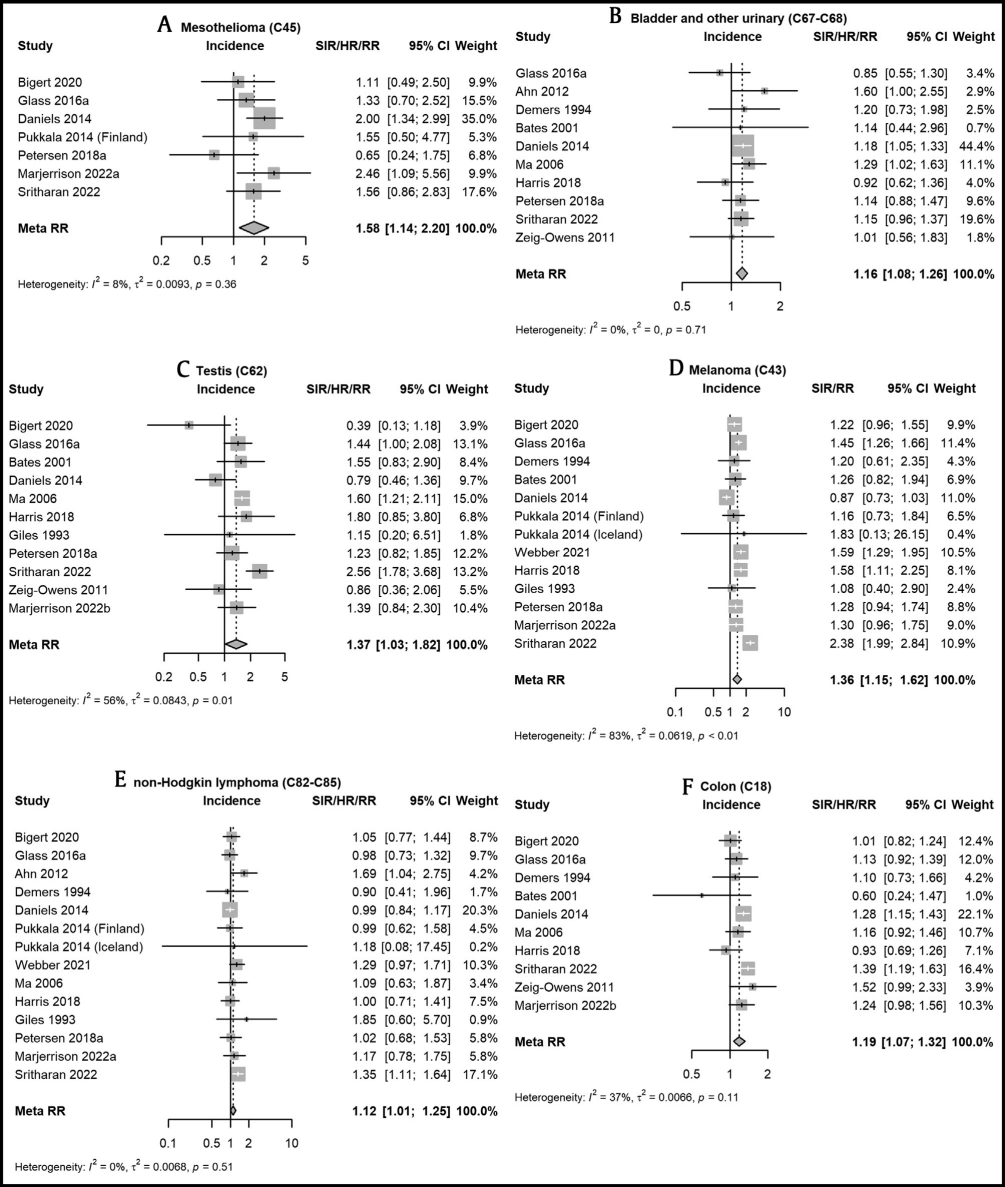
Forest plots of individual study results and meta-rate ratios for select cancers among male career firefighters compared to a general, uniformed service, or working population referent.^†^ † Random-effects models were used with the restricted maximum-likelihood estimator. Hartung-Knapp-Sidik-Jonkman (HKSJ) adjustments and an ad hoc variance correction were used to calculate confidence intervals for summary estimates. Calculated study intervals may differ from reported values due to differences in variance estimation methods. Presented cancer sites were chosen for having positive associations and relevant exposures related to the firefighting occupation.

Forest plots for the main analysis of cancer incidence are shown in Fig. 1 (for select cancer sites) and in Suppl. Fig. 3 for the others. Compared to general, uniformed service (e.g., police or military), or working referent populations (Table 2), positive associations were observed between ever-employment as a firefighter and the incidence of mesothelioma, melanoma of the skin, cancers of the testis, thyroid, prostate, colon, bladder, and NHL. In contrast, an inverse association was observed with the incidence of lung cancer. Of the 13 cancer outcomes assessed, six presented with significant between-study heterogeneity. There was little evidence of elevated incidence for cancers of the stomach, kidney, or brain and nervous system.

**Table 2:** Meta-rate ratios (mRR) for select cancers among male career firefighters compared to a general, uniformed service, or working population referent

Outcome	Studies∗ (n)	mRR† (95% CI)	I^2^‡ (%)	Q *p*-value	τ^2^
**Incidence (SIR, RR, HR)**					
All cancers (C00-C95)	14	1.05 (0.99–1.11)	87	<0.01	0.008
Stomach (C16)	12	1.00 (0.87–1.15)	33	0.12	0.002
Colon (C18)	10	1.19 (1.07–1.32)	37	0.11	0.007
Lung (C33-C34)	14	0.85 (0.75–0.96)	78	<0.01	0.032
Melanoma (C43)	12	1.36 (1.15–1.62)	83	<0.01	0.062
Mesothelioma (C45)	7	1.58 (1.14–2.20)	8	0.36	0.009
Prostate (C61)	14	1.21 (1.12–1.32)	81	<0.01	0.015
Testis (C62)	11	1.37 (1.03–1.82)	56	0.01	0.084
Kidney (C64-C66)	12	1.09 (0.92–1.29)	55	0.01	0.035
Bladder (C67-C68)	10	1.16 (1.08–1.26)	0	0.71	0
Brain and nervous (C47, C70-C72)	11	1.01 (0.86–1.18)	5	0.40	0.003
Thyroid (C73)	10	1.28 (1.02–1.61)	40	0.09	0.055
Non-Hodgkin lymphoma (C82-C85)	13	1.12 (1.01–1.25)	0	0.51	0.007
**Mortality (SMR, RR)**§					
All cancers (C00-C95)	18	0.96 (0.88–1.06)	87	<0.01	0.026
Stomach (C16)	13	1.05 (0.87–1.28)	41	0.06	0.045
Colon (C18)	9	1.03 (0.78–1.37)	63	<0.01	0.079
Lung (C33-C34)	12	0.96 (0.86–1.06)	55	0.01	0.008
Melanoma (C43)	4	1.05 (0.48–2.30)	0	0.43	0.093
Mesothelioma (C45)	3	1.75 (0.83–3.69)	0	0.56	0
Prostate (C61)	11	1.07 (0.95–1.20)	30	0.16	0
Kidney (C64-C66)	9	1.10 (0.66–1.83)	53	0.03	0.199
Bladder (C67-C68)	9	1.22 (0.70–2.11)	67	<0.01	0.267
Brain and nervous (C47, C70-C72)	11	1.33 (0.98–1.79)	53	0.02	0.098
Thyroid (C73)	4	1.90 (0.36–10.00)	58	0.07	0.671
Non-Hodgkin lymphoma (C82-C85)	5	1.20 (1.03–1.40)	0	0.74	0

Abbreviations: mRR, meta-rate ratio; CI, confidence interval; SIR, standardized incidence ratio; RR, rate ratio; HR, hazard ratio; SMR, standardized mortality ratio.

∗ Results from the studies by Daniels (2014) and Pinkerton (2020) include a small number of females. Petersen et al. (2018a) includes part-time/volunteer firefighters for kidney, stomach, thyroid, brain, and mesothelioma. Some results from overlapping study populations are excluded.

† Random-effects models were used with between-study variance estimated using the restricted maximum-likelihood estimator. Hartung-Knapp-Sidik-Jonkman (HKSJ) adjustments and an ad hoc variance correction (using wider confidence intervals) were used to calculate confidence intervals.

‡ See Fig. 1 for individual study results and generic inverse variance meta-analysis statistics. The variance of individual study estimates is based on the reported confidence interval bounds and may differ from estimates obtained using exact methods when there are few cases.

§ Outcomes with fewer than three available studies were not meta-analyzed.

For cancer mortality outcomes, there was a positive association between ever-employment as a firefighter and NHL. This estimate was slightly greater in magnitude than that observed for NHL incidence. Neither model exhibited significant residual heterogeneity. There were five studies in the mortality analysis for NHL, with nearly all weight (89.4%) given to a single study of US firefighters [13]. Positive associations were also observed for mortality from mesothelioma and cancers of the bladder, thyroid, and brain, although the CIs were wide for most of these outcomes.

### 3.2 Duration of employment

There were nine studies included in the meta-analysis of the association between duration of employment and cancer incidence (Table 3) [14,16,22,25,27,33,37,39,41]. A positive association was observed in the >20-year duration category for colon cancer incidence, although duration subgroups did not significantly differ (*p* = 0.67). Mesothelioma incidence was suggested to be inversely associated with employment duration, although estimates were statistically imprecise. Results from the meta-regression of individual study effect estimates for employment duration suggested that the most positive linear trends were for cancers of colon and brain (Suppl. Table E), although results were also imprecise. Overall, there was little evidence of a positive exposure-response association between employment duration and cancer incidence for any cancer site.

**Table 3:** Sensitivity analyses of Table 2 results with the use of alternative referent populations and application of bias and age restrictions

Outcome	Sensitivity analysis∗ mRR (95% CI) No. of studies, I^2^ (%), Q *p*-value																				
	Table 2 results			Priority to uniformed service or working comparison			Restricted to general population comparisons			Restricted to uniformed service comparisons			Restricted to mean age ≥55 years at follow-up end or >20 years follow-up			Excludes “major” concern for healthy worker bias			Excludes “major” concern for surveillance bias		
**Incidence**																					
All cancers (C00-C95)	1.05 (0.99–1.11)			1.03 (0.98–1.07)			1.03 (0.98–1.09)			1.03 (0.99–1.07)			1.09 (1.01–1.17)			1.04 (0.97–1.12)			1.08 (1.02–1.14)		
	14	87	<0.01	14	79.6	<0.01	13	81.3	<0.01	4	0.0	0.81	6	83.5	<0.01	11	89.2	<0.01	9	77.1	<0.01
Stomach (C16)	1.00 (0.87–1.15)			1.01 (0.86–1.18)			1.02 (0.88–1.18)			0.90 (0.29–2.80)			1.05 (0.84–1.30)			0.96 (0.79–1.17)			1.02 (0.81–1.28)		
	12	33	0.12	12	39.6	0.08	11	30.2	0.16	3	57.3	0.10	5	23.5	0.27	9	35.0	0.14	7	20.9	0.27
Colon (C18)	1.19 (1.07–1.32)			1.13 (1.02–1.26)			1.16 (1.04–1.29)			—			1.24 (1.01–1.52)			1.23 (1.08–1.40)			1.13 (0.96–1.33)		
	10	37	0.11	10	34.4	0.13	9	26.0	0.21				4	51.3	0.10	7	33.3	0.17	7	47.5	0.08
Lung (C33-C34)	0.85 (0.75–0.96)			0.90 (0.81–1.00)			0.85 (0.74–0.97)			1.05 (0.89–1.25)			0.92 (0.80–1.05)			0.89 (0.79–1.00)			0.88 (0.81–0.94)		
	14	78	<0.01	14	70.0	<0.01	13	79.0	<0.01	4	0.0	0.51	6	75.1	<0.01	11	74.9	<0.01	9	0.0	0.75
Melanoma (C43)	1.36 (1.15–1.62)			1.19 (1.05–1.35)			1.27 (1.11–1.46)			1.05 (0.90–1.21)			1.32 (0.96–1.80)			1.33 (1.06–1.69)			1.45 (1.20–1.75)		
	12	83	<0.01	12	54.0	0.01	11	61.6	<0.01	4	0.0	0.97	6	90.8	<0.01	9	86.3	<0.01	9	71.7	<0.01
Mesothelioma (C45)	1.58 (1.14–2.20)			1.64 (1.12–2.42)			1.54 (0.99–2.38)			—			1.61 (1.07–2.44)			1.74 (1.10–2.75)			1.52 (1.08–2.14)		
	7	8	0.36	7	17.9	0.29	6	23.6	0.26				6	19.0	0.29	5	22.2	0.27	5	0.0	0.72
Prostate (C61)	1.21 (1.12–1.32)			1.12 (1.06–1.18)			1.19 (1.10–1.29)			1.08 (0.89–1.32)			1.16 (1.03–1.30)			1.19 (1.09–1.30)			1.22 (1.12–1.33)		
	14	81	<0.01	14	30.9	0.12	13	75.1	<0.01	4	55.0	0.08	6	84.5	<0.01	11	73.6	<0.01	9	63.0	<0.01
Testis (C62)	1.37 (1.03–1.82)			1.36 (1.09–1.69)			1.31 (1.04–1.64)			—			1.19 (0.53–2.63)			1.49 (1.11–2.02)			1.54 (0.99–2.39)		
	11	56	0.01	11	29.6	0.16	10	25.9	0.21				5	80.3	<0.01	8	53.3	0.04	7	55.0	0.04
Kidney (C64-C66)	1.09 (0.92–1.29)			1.07 (0.91–1.25)			1.04 (0.89–1.22)			1.01 (0.60–1.70)			1.18 (0.89–1.57)			1.20 (0.90–1.46)			1.11 (0.84–1.45)		
	12	55	0.01	12	37.9	0.09	11	35.0	0.12	4	47.4	0.13	5	62.8	0.03	9	42.5	0.08	7	57.3	0.03
Bladder (C67-C68)	1.16 (1.08–1.26)			1.13 (1.01–1.26)			1.17 (1.07–1.28)			1.02 (0.74–1.39)			1.17 (1.06–1.28)			1.18 (1.09–1.28)			1.08 (0.92–1.27)		
	10	0	0.71	10	8.3	0.37	9	0.0	0.62	3	0.0	0.42	3	0.0	0.96	8	0.0	0.79	5	0.0	0.64
Brain and nervous (C47-C70-C72)	1.01 (0.86–1.18)			0.97 (0.84–1.12)			0.97 (0.82–1.13)			0.96 (0.73–1.26)			1.07 (0.87–1.31)			1.06 (0.88–1.27)			1.08 (0.89–1.31)		
	11	5	0.40	11	0.0	0.56	10	0.0	0.50	3	0.0	0.80	5	0.0	0.44	9	6.6	0.38	7	0.0	0.57
Thyroid (C73)	1.28 (1.02–1.61)			1.17 (0.89–1.54)			1.31 (1.01–1.69)			1.17 (0.30–4.50)			1.04 (0.78–1.40)			1.15 (0.93–1.43)			1.16 (0.88–1.53)		
	10	40	0.09	9	37.2	0.12	9	43.2	0.08	3	70.9	0.03	4	0.0	0.68	8	0.0	0.54	5	0.0	0.96
Non-Hodgkin lymphoma (C82-C85)	1.12 (1.01–1.25)			1.07 (0.97–1.18)			1.07 (0.97–1.18)			1.13 (0.91–1.40)			1.11 (0.96–1.28)			1.13 (0.99–1.29)			1.12 (0.98–1.29)		
	13	0	0.51	13	0.0	0.88	12	0.0	0.80	4	0.0	0.69	6	3.9	0.40	10	3.5	<0.01	8	0.0	0.63
**Mortality**†																					
All cancers (C00-C95)	0.96 (0.88–1.06)			0.97 (0.88–1.06)			0.95 (0.86–1.05)			—			1.04 (0.97–1.12)			1.01 (0.94–1.07)			0.96 (0.87–1.05)		
	18	87	<0.01	18	87.2	<0.01	16	88.6	<0.01				7	71.7	<0.01	15	77.5	<0.01	17	87.8	<0.01
Stomach (C16)	1.05 (0.87–1.28)			1.05 (0.87–1.28)			0.97 (0.82–1.15)			—			1.14 (0.90–1.43)			1.12 (0.96–1.30)			1.00 (0.85–1.18)		
	13	41	0.06	13	41.1	0.06	11	14.2	0.31				7	35.4	0.16	11	13.0	0.32	12	18.1	0.27
Colon (C18)	1.03 (0.78–1.37)			1.03 (0.78–1.37)			1.05 (0.75–1.48)			—			0.87 (0.51–1.49)			0.90 (0.67–1.21)			0.98 (0.70–1.36)		
	9	63	<0.01	9	62.6	<0.01	7	48.0	0.07				4	49.2	0.12	7	40.5	0.12	8	55.1	0.03
Lung (C33-C34)	0.96 (0.86–1.06)			0.96 (0.86–1.07)			0.95 (0.84–1.07)			—			0.97 (0.87–1.07)			0.98 (0.90–1.07)			0.96 (0.86–1.06)		
	12	55	0.01	12	54.3	0.01	10	59.0	0.01				6	51.1	0.07	11	39.5	0.09	12	54.6	0.01
Melanoma (C43)	1.05 (0.48–2.30)			1.05 (0.48–2.30)			1.28 (0.45–3.66)			—			—			1.05 (0.48–2.30)			1.05 (0.48–2.30)		
	4	0	0.43	4	0.0	0.43	3	0.0	0.47							4	0.0	0.43	4	0.0	0.43
Mesothelioma (C45)	1.75 (0.83–3.69)			1.75 (0.83–3.69)			—			—			—			1.75 (0.83–3.69)			1.75 (0.83–3.69)		
	3	0	0.56	3	0.0	0.56										3	0.0	0.56	3	0.0	0.56
Prostate (C61)	1.07 (0.95–1.20)			1.06 (0.94–1.19)			1.08 (0.96–1.22)			—			1.01 (0.76–1.35)			1.07 (0.95–1.21)			1.08 (0.97–1.21)		
	11	30	0.16	11	26.6	0.19	9	23.0	0.24				7	53.1	0.05	10	34.4	0.13	10	15.2	0.30
Kidney (C64-C66)	1.10 (0.66–1.83)			1.10 (0.66–1.83)			1.07 (0.58–1.99)			—			0.92 (0.59–1.44)			1.08 (0.60–1.94)			1.10 (0.66–1.83)		
	9	53	0.03	9	52.7	0.03	8	58.6	0.02				6	33.1	0.19	8	58.5	0.02	9	52.7	0.03
Bladder (C67-C68)	1.22 (0.70–2.11)			1.23 (0.70–2.15)			1.34 (0.74–2.43)			—			0.95 (0.67–1.34)			1.07 (0.63–1.81)			1.22 (0.70–2.11)		
	9	67	<0.01	9	66.8	<0.01	8	67.3	<0.01				4	30.5	0.23	8	57.0	0.02	9	66.9	<0.01
Brain and nervous (C47-C70-C72)	1.33 (0.98–1.79)			1.26 (0.94–1.67)			1.37 (0.98–1.93)			—			1.58 (0.96–2.60)			1.27 (0.93–1.74)			1.33 (0.98–1.79)		
	11	53	0.02	11	39.4	0.09	10	57.2	0.01				5	71.1	0.01	10	52.3	0.03	11	52.8	0.02
Thyroid (C73)	1.90 (0.36–10.0)			1.90 (0.36–10.0)			1.76 (0.08–39.1)			—			—			1.90 (0.36–10.0)			1.90 (0.36–10.0)		
	4	58	0.07	4	58.4	0.07	3	72.1	0.03							4	58.4	0.07	4	58.4	0.07
Non-Hodgkin lymphoma (C82-C85)	1.20 (1.03–1.40)			1.18 (1.00–1.40)			1.20 (1.03–1.40)			—			1.21 (1.04–1.41)			1.20 (1.03–1.40)			1.20 (1.03–1.40)		
	5	0	0.74	5	0.0	0.67	5	0.0	0.74				4	0.0	0.77	5	0.0	0.74	5	0.0	0.74

Abbreviations: mRR, meta-rate ratio; CI, confidence interval.

∗ Random-effects models were used with between-study variance estimated using the restricted maximum-likelihood estimator. Hartung-Knapp-Sidik-Jonkman (HKSJ) adjustments and an ad hoc variance correction (using wider confidence intervals) were used to calculate confidence intervals. The variance of individual study estimates is based on the reported confidence interval bounds and may differ from estimates obtained using exact methods when there are few cases.

† Outcomes with fewer than three available studies were not meta-analyzed.

For mortality outcomes, there were 10 studies included in meta-analysis of results for employment duration (Suppl. Table D) [16,17,29,32,40,42,44,[48], [49], [50]]. Subgroup analysis revealed significant residual heterogeneity in models of all cancers combined and brain cancer. This heterogeneity persisted in meta-regression indicating that the exposure-response association did not explain the between-study variance. In contrast, adding the exposure covariate to the model for stomach cancer mortality reduced the residual variance by about 43%. There was evidence of a positive trend in stomach cancer mortality, although the slope parameter was not significant (*p* = 0.12) (Suppl. Table E). There was little evidence of a positive association between employment duration and cancer mortality among the remaining cancer sites.

### 3.3 Sensitivity analyses

Results from sensitivity analyses are shown in Table 3. Meta-estimates giving priority to results using uniformed service comparison populations were consistent with the main analysis but tended to be slightly attenuated toward the null value. An exception was the positive association for mesothelioma incidence, which was slightly elevated away from the null compared to the main analysis. Estimates from analyses restricted to results using a general population referent did not differ markedly from the main analysis, which reflects that most studies used general population reference groups. In contrast, meta-estimates shifted downward in analyses restricted to results using only a uniformed service referent population for all cancer sites except lung cancer and NHL incidence. This suggested that the choice of reference group may possibly explain some heterogeneity in analyses incorporating studies with different reference populations.

Overall, most estimates (60%) increased slightly in magnitude after restricting to studies with mean age at study end ≥55 years or length of follow-up >20 years. This increase in the magnitude of estimates is consistent with a reduction in a healthy worker hire bias expected from studies of younger cohorts followed more closely to the time of hire. It also suggests that the 55–70-year age period may be the most relevant for observing positive associations between firefighting and cancer. Notable exceptions were estimates for prostate (incidence and mortality), testis, and thyroid cancer, which attenuated toward the null in studies with older attained age or longer periods of follow-up. Excluding studies with “major” concern for healthy worker bias did not meaningfully change most estimates. However, estimates for mesothelioma and testis cancer incidence increased further away from the null, while those for thyroid incidence decreased. Similar findings were observed with the exclusion of studies with “major” concern for surveillance bias, with estimates for melanoma and testis cancer incidence increasing further from the null, and those for thyroid attenuating downward.

## 4 Discussion

In the 35 epidemiological cohort studies included in this review, there was evidence of positive associations between occupational exposure as a firefighter and cancer incidence for several sites, including bladder, testis, prostate, thyroid, and colon cancer, as well as mesothelioma, NHL, and melanoma. Associations for bladder cancer and NHL were modest in magnitude. For mortality outcomes, associations were attenuated compared with incidence outcomes for prostate, colon cancer, and melanoma, while they were similar or greater in magnitude for bladder, lung cancer, NHL, and mesothelioma. Since the most recent meta-analysis on cancer in firefighters [3], three new cohort studies [[20], [21], [22],^24^] and two cohorts with extended follow-up [13,25] have been published that were included in this review. Our results from comparable analyses were consistent with those previously reported and suggested more strongly positive associations for the incidence of testis, colon, and prostate cancer, as well as for mesothelioma and melanoma. Applying a causal interpretation to our findings requires additional considerations regarding the influence of bias and the plausibility of exposures in the occupation to cause specific cancer types over time. Studies of cancer in firefighters are subject to substantial influence from medical surveillance bias, healthy worker hire and survivor bias, and confounding, which we sought to evaluate in sensitivity analyses. Additionally, firefighters can be exposed to various and complex mixtures of carcinogenic hazards during fire and non-fire events. The types of activities performed, use of personal protective equipment, and composition of exposures in the occupation have changed significantly over time and differ by region and type of fire suppression activity (e.g., wildland, structural, and vehicular). Despite the large volume of epidemiological research on this topic, these factors make causal assessments of the associations observed in meta-analysis of human cancer studies challenging.

### 4.1 Respiratory system cancer

We observed an elevated risk of mesothelioma among firefighters, but no evidence of higher risk for cancer of the lung, including the trachea and bronchus. The positive association observed for mesothelioma incidence was strong in magnitude relative to other summary estimates, and the mRR estimate exhibited little heterogeneity. The combined information from seven cohort studies was consistent in showing a positive association, except for one study from Denmark which was based on only four cases and a study population consisting of a high proportion of part-time and volunteer firefighters [26]. Removing this study from the meta-analysis increased the mRR from 1.58 to 1.70 and reduced the heterogeneity to 0%. The positive association remained similar in magnitude in sensitivity analyses using alternative referent populations and applying age period and bias restrictions. Although analyses by duration of employment showed an inverse monotonic association, these results were only based on three studies and estimates were highly imprecise. Structural firefighters may be exposed to asbestos during multiple activities that can disturb building materials containing asbestos, such as fire suppression, overhaul, rescue, and recovery. Exposure could also occur from the resuspension of asbestos fibers from contamination on apparatus and firefighting gear. Confounding due to asbestos exposure outside of the firefighting occupation is unlikely to explain the magnitude and consistency of results for mesothelioma across studies. Findings for mesothelioma have only recently become observable with adequate validity due to the unavailability of diagnostic codes for mesothelioma in the ICD before the introduction of the 10^th^ revision in 1999. While cancer incidence studies with ICD-10 codes capable of capturing mesothelioma diagnoses have recently become available, the lack of a cause-of-death code before 1999 may have obscured the risk of this cancer in older cohort studies ascertaining mortality outcomes.

Despite firefighters being potentially exposed to several known human lung carcinogens, including components of smoke (e.g., soot) and diesel engine exhaust, there was no evidence in any analyses that employment as a firefighter was positively associated with either lung cancer incidence or mortality, although mortality findings were closer to the null than incidence findings. These findings suggest that the inhalation of combustion products may not be sufficient to cause an increased risk of lung cancer among most firefighters (or to overcome downward biases), possibly due to exposure reduction controls that are effective in reducing exposures, such as self-contained breathing apparatus. Alternatively, factors that could obscure a positive association include the healthy worker hire bias and the potential for negative confounding due to tobacco smoking in studies with more recent calendar periods of follow-up. Available information on tobacco smoking prevalence in firefighters is sparse and restricted primarily to the USA, although studies suggest that firefighters have a lower prevalence of smoking than the general population, with one US study observing a trend of lower smoking since at least the early 1990s [[51], [52], [53], [54]]. No studies included in the meta-analysis controlled directly for smoking status. The attenuation of the risk deficit when giving priority to uniformed service or working comparison groups and when restricting to periods of longer follow-up also supports a potential role of healthy worker biases. The mRR for lung cancer incidence using uniformed service comparison groups was 20 percentage points greater than that using general populations, although it still showed little evidence of a positive association. Consistent with our meta-analysis results, findings from the pooled international SYNERGY case-control study showed no increased risk of lung cancer overall or by histological cell type among firefighters with or without adjustment for smoking [12].

### 4.2 Genitourinary cancer

Positive associations were observed for bladder, testis, and prostate cancer incidence, but there was little evidence of elevated risk for cancer of the kidney. The positive association for bladder cancer incidence was modest in magnitude, although the estimate was statistically precise, with little heterogeneity (I^2^ = 0%). Results for bladder cancer mortality were also consistent with the incidence results in showing a modest positive association, despite the mortality results exhibiting lower statistical precision. Results from two studies of firefighters in Norway and Sweden with long periods of follow-up were excluded from the bladder cancer analyses because they used broader case definitions that included cancers of the urinary tract (bladder, other urinary, ureter, and renal pelvis combined; ICD-10 C65-C68). However, both studies reported positive results, and including them in the meta-analysis yielded an identical mRR estimate of 1.16 (95% CI 1.08–1.24, I^2^ = 0%). Although results did not indicate a positive association for bladder cancer incidence with increasing duration of employment, duration analyses may be biased downward due to healthy worker survivor bias, assuming that firefighters employed for longer durations receive less exposure to fire hazards with greater seniority, and that less healthy workers leave the occupation after shorter durations due to the effects of exposure or diminishing health status. In a pooled US study of municipal firefighters [13], a strong indication of confounding by employment duration was observed in internal analyses of the association between exposed days and bladder cancer mortality, where the estimate shifted from a negative to a positive association after adjustment for employment duration.

Firefighters can be frequently exposed to combustion products from fires, including soot and PAHs, as well as diesel engine exhaust, which are known or suspected causes of bladder cancer in humans [55]. Although these agents can cause lung cancer as well, differences in the route of exposure (e.g., ingestion/absorption versus inhalation) and metabolism of these agents in the urinary tract may impart risks specific to the bladder. Findings among aluminum production workers (who are primarily exposed to PAHs) showed a similar pattern of stronger associations with bladder than with lung cancer [56]. Tobacco smoking is not expected to be a positive confounder of the observed associations for bladder cancer given the inverse associations we observed for lung cancer and evidence suggesting reduced smoking prevalence among firefighters compared to the general population [52]. Further, some evidence from studies of bladder cancer among firefighters with known smoking status indicates that positive associations may persist after adjustment for smoking [57,58].

The positive association observed for testicular cancer incidence in the main analysis was greater in magnitude (mRR 1.37) than the associations observed for all other cancer sites aside from mesothelioma, although the estimate exhibited high heterogeneity (I^2^ = 56%). The association was attenuated in the sensitivity analysis restricting to studies with older age/length of follow-up, suggesting that the increased risk in firefighters is greatest during younger age periods, which is when testicular cancer is most commonly diagnosed in the general population (<35 years) [59]. Standardized screening methods for testicular cancer are not available, and most tumors are found by self- or medical exam. Based on tumor behavior and progression, early detection is not likely to explain the observed excess risk. Overall, no environmental or occupational exposures have been established as known causes of testicular cancer. However, firefighters may be exposed to some compounds with "limited" evidence of human testicular carcinogenicity, including perfluorooctanoic acid (PFOA). Aqueous film-forming foams (AFFF) are fire suppressants used to fight flammable liquid fires in training facilities, vehicles, ships, and aircraft, and they contain PFOA (or other PFAS) and/or similar compounds. However, the extent of AFFF exposure among firefighters examined in the included studies is unclear.

Positive associations were observed with prostate cancer incidence, although the mRR exhibited high heterogeneity (I^2^ = 81%), and associations were attenuated when using mortality as an outcome. Associations with prostate cancer incidence were also attenuated by more than 10% when restricting the meta-analysis to studies using other uniformed service populations as a comparison group. Firefighters may benefit from increased medical surveillance and more frequent cancer screenings than the general population due to greater access to routine medical assessments and uptake of cancer prevention initiatives. Such surveillance can make it more likely for cancers that would not otherwise have been identified, or detected at a later stage, to be detected in firefighters, even for cancer types that do not have broad population-based screening programs. The introduction of prostate-specific antigen testing in the 1980s has led to an increase in the incidence of prostate cancer diagnoses in the general population, and this trend may have been accentuated in populations with greater medical screening. A study of all prostate cancer cases diagnosed over a 57-year period in Norway showed that firefighters were diagnosed with prostate cancer at a younger age and had better prognostic markers at diagnosis compared to other men in the general population, and that the difference was most pronounced during the most recent decade of observation (2007–2017) [60]. Men in other uniformed service occupations with regular health screenings showed similar results to firefighters. Our exclusion of studies with “major” concern for surveillance bias in sensitivity analysis may not have been sufficient to fully account for the positive influence of screening on our prostate cancer results.

### 4.3 Other cancers

Cancers in other organ systems also showed evidence of positive associations among firefighters, including melanoma of the skin, NHL, and colon cancer. A positive association of moderate magnitude was observed for the incidence of melanoma, although the mRR exhibited considerable heterogeneity, and the estimate was attenuated to a null association when using uniformed service populations as the comparison group. Melanoma was one of few cancer sites that showed positive and statistically precise associations in all three categories of duration of employment. While firefighters can be occupationally exposed to agents known to cause melanoma, including solar radiation and PCBs [55], sources of confounding could contribute to the observed findings, including differences in the distribution of non-firefighting-related sun exposure and skin tone between firefighters and comparison groups. Information on race was available in only two US studies and suggested a greater prevalence of White race among firefighters than the general population [61,62]. Further, medical surveillance bias could also explain the excess risk in firefighters, as skin cancer screening and secondary prevention campaigns have been shown to increase the frequency of melanoma diagnoses [63]. In contrast to melanoma and non-melanoma skin cancers have soot exposure as an established cause [55], although positive results for non-melanoma skin cancer were only observed in one of the four studies that reported results for both skin cancer types [25].

A modest positive association was observed for both NHL incidence and mortality outcomes, and both estimates exhibited little heterogeneity (I^2^ = 0%). The associations persisted in all sensitivity analyses; however, individual studies were inconsistent in showing positive findings. NHL was among the cancer types first reported to be positively associated with firefighting in a prior meta-analysis in 2006 [5]. Firefighters can be exposed to agents that are either known or suspected causes of NHL, including exposure to PAHs in combustion products and benzene [55]. Interpretation of findings for NHL is complicated by the heterogenous subtypes of the disease with distinct etiologic characteristics and evolving diagnostic criteria that have changed the classification of cancer across time and between studies. The distribution of NHL subtypes can vary geographically and may influence discrepant results between studies in different countries. Changing definitions of NHL over time may have led to some inconsistency in results, particularly if there is heterogeneity in the association with firefighting by tumor subtype. Furthermore, confounding patterns for NHL may vary by subtype (e.g., alcohol consumption appears inversely related to some forms of NHL) [64].

Colon cancer incidence was observed to be in excess among firefighters, although there was no evidence of positive associations with mortality. The mRR was modest in magnitude with some between-study heterogeneity (I^2^ = 37%), and there were too few studies available for reliable estimates of the association with the duration of employment. Because of the positive associations with colon cancer incidence and not mortality, surveillance bias via greater screening among firefighters may partially explain observed findings. Firefighters are required to maintain a high level of physical fitness to enter their profession, and physical activity has been associated with a decreased risk of colon cancer [65], although little is known about this and other non-occupational risk factors for colon cancer among firefighters.

Thyroid and brain and nervous system cancer also showed some evidence of being positively associated with occupational exposure as a firefighter. Excess risk was observed for thyroid cancer incidence, although results were attenuated in most sensitivity analyses. Thyroid cancer may be particularly vulnerable to surveillance bias due to overdiagnosis of occult lesions, which has been demonstrated in the cohort of firefighters exposed to the collapse of the World Trade Center in the USA, who receive extensive medical surveillance [66]. The mRR was substantially influenced by the inclusion of one study from this cohort which reported the highest effect estimate for thyroid cancer even after the authors applied a surveillance bias adjustment [23]. A positive association was also observed for brain and nervous system cancer mortality, although findings for incidence outcomes were null. The duration of employment results for mortality outcomes showed suggestive elevations in all duration categories, although estimates were statistically imprecise. Results for brain and nervous system cancer are consistent with findings from the previous meta-analysis [3], and potential explanations for the positive findings in older mortality-based studies are unclear.

Strengths of this work, aside from the incorporation of new and updated results from cohort studies, are the meta-analysis of duration of employment as a firefighter and sensitivity analyses using different referent populations, age/length of follow-up restrictions, and our tailored bias assessment exercise for major sources of bias applicable to studies on the topic. These additional analyses complement results from previous systematic reviews and meta-analyses of cancer among firefighters [[3], [4], [5],^67^]. Despite these strengths, we could not meta-analyze internal comparison results for cumulative exposure to firefighting activities, such as number of exposed days, number of fire responses, or types of fires fought, as few studies conducted such analyses and those that did used inconsistent metrics. As a result, inferences from this work are largely based on results from analyses limited to ever-employment and duration of employment in the occupation. The reviewed studies are limited by challenges in exposure assessment; medical surveillance bias; adjustment for individual confounding factors, such as tobacco consumption, healthy worker biases; and short length of follow-up.

## 5 Conclusion

Among studies included in this meta-analysis, positive associations were observed between employment as a firefighter and mesothelioma and cancer of the bladder, which could plausibly be related to exposures in the occupation, although the association for bladder cancer was modest in magnitude. Positive associations were also observed for cancer of the prostate, testis, and colon, as well as melanoma and NHL. However, for the latter group of cancer sites, findings were either inconsistent across individual studies and sensitivity analyses, or sources of bias were more likely to have partially or fully explained the positive results.

The carcinogenic exposures inherent in the firefighting occupation make exposure reduction a critical imperative of future research efforts. Firefighters in several countries benefit from existing presumptive workers' compensation policies for cancer, medical health screening programs, workplace cancer prevention and awareness programs, and exposure reduction controls. Given this context, future research should focus on providing evidence needed to better inform existing prevention efforts and transition to a greater emphasis on primary prevention. Additional etiologic studies of cancer in firefighters require more sophisticated designs with detailed and harmonized exposure metrics, mechanistic endpoints, and a focus on understudied populations, such as women, wildland firefighters, and firefighters from low- and middle-income countries. Nonetheless, primary prevention through reducing firefighters exposure to known or suspected carcinogenic hazards should be the overarching aim of future research in the field.

## Disclaimer

Where authors are identified as personnel of the International Agency for Research on Cancer/World Health Organization or the National Institute for Occupational Safety and Health, Centers for Disease Control and Prevention, the authors alone are responsible for the views expressed in this article and they do not necessarily represent the decisions, policy, or views of the International Agency for Research on Cancer/World Health Organization or the National Institute for Occupational Safety and Health, Centers for Disease Control and Prevention.

## Funding

This work was supported by the 10.13039/100000002National Institutes of Health, including the 10.13039/100000054National Cancer Institute and 10.13039/100000066National Institute of Environmental Health Sciences [NIH-NCI U01CA033193].

## Conflicts of interest

All authors declare that they have no conflicts of interest related to this work. All authors declare that they have no financial or professional interest that would constitute a conflict of interest for the scientific integrity or the interpretation of any data related to this research. This includes employment and consulting activities, individual and institutional research support, and other financial or non-financial interests (e.g., public statements and positions related to the subject of the research).
