# Supplementary material for: Firefighting and Cancer: A Meta-analysis of Cohort Studies in the Context of Cancer Hazard Identification
Source: Saf Health Work. 2023 Mar 7;14(2):141–52. doi: 10.1016/j.shaw.2023.02.003 (PMC10300491; doi:10.1016/j.shaw.2023.02.003)
Supplement: Multimedia component 1 [file mmc1.docx]

Supplemental Table A. Search terms and databases used to identify epidemiological studies for inclusion in the review

| Database | Search terms |
| --- | --- |
| PubMed^*^ | (firefighters[mh] OR firefight*[tw] OR firemen[tw] OR fireman[tw] OR fire brigade*[tw] OR fire rescue[tw] OR fire crew*[tw] OR firewomen[tw] OR (fire* AND fight*))  AND  ((neoplasm* OR carcinogen* OR malignan* OR tumor OR tumors OR tumour OR tumours OR cancer OR cancers)  OR  (mortality[mh] or death[mh] or mortalit*[tw] or mortalit*[sh] or death*[tw])) |
| Web of Science | TS = (firefight* OR firem?n OR fire brigade* OR fire rescue* OR fire crew* OR (fire near/3 fight*))  AND  TS = (neoplasm* OR carcinogen* OR malignan* OR tumo$r* OR cancer*) |
| Embase | ('fire fighter'/exp OR 'fire fighter' OR firefight* OR firem*n OR 'fire brigade*' OR 'fire rescue*' OR 'fire crew*' OR (fire NEAR/3 fight*))  AND  (neoplasm* OR carcinogen* OR malignan* OR tumo?r* OR cancer*) |

* The explicit search terms for mortality were used to target occupational mortality and mortality surveillance studies that did not specifically mention cancer in their MeSH heading, title, or abstract.

Supplemental Figure 1. Results and disposition of literature search through 13 June 2022


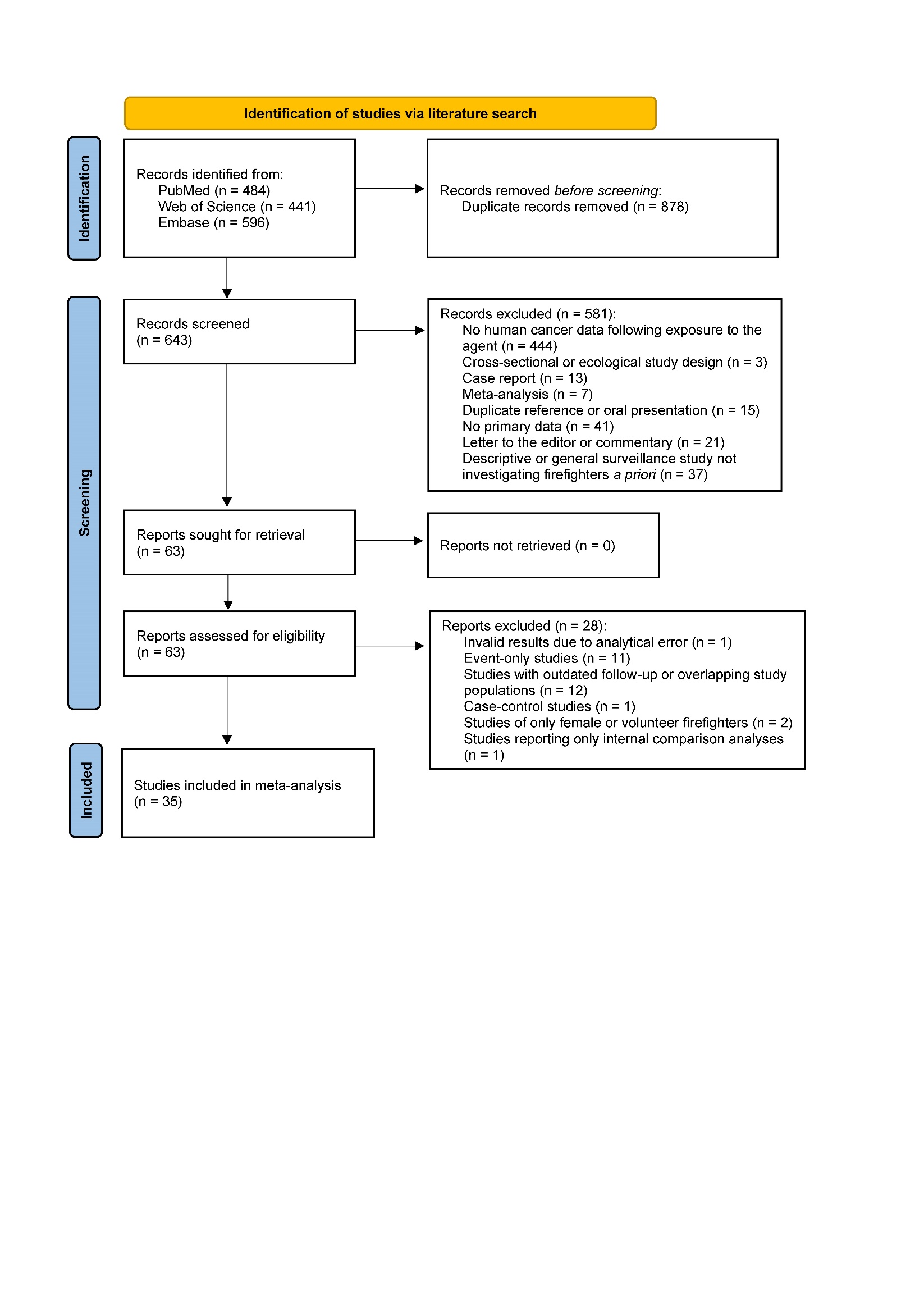


Supplemental Table B. Studies included in Table 2 meta-analyses for each cancer site (incidence outcomes)

| Study | Cancer site | | | | | | | | | | | | |
| --- | --- | --- | --- | --- | --- | --- | --- | --- | --- | --- | --- | --- | --- |
|  | All-cancer | Stomach | Colon | Lung | Melanoma | Meso. | Prostate | Testis | Kidney | Bladder | Brain | Thyroid | NHL |
| Ahn 2012 | Yes | Yes | - | Yes | - | - | Yes | - | Yes | Yes | Yes | Yes | Yes |
| Bates 2001 | Yes | Yes | Yes | Yes | Yes | - | Yes | Yes | Yes | Yes | Yes | - | - |
| Bigert 2020 | Yes | Yes | Yes | Yes | Yes | Yes | Yes | Yes | Yes | - | Yes | - | Yes |
| Daniels 2014 | Yes | Yes | Yes | Yes | Yes | Yes | Yes | Yes | Yes | Yes | Yes | Yes | Yes |
| Demers 1994 | Yes | Yes | Yes | Yes | Yes | - | Yes | - | Yes | Yes | Yes | Yes | Yes |
| Giles 1993 | Yes | - | - | Yes | Yes | - | Yes | Yes | - | - | - | - | Yes |
| Glass 2016 | Yes | Yes | Yes | Yes | Yes | Yes | Yes | Yes | Yes | Yes | Yes | Yes | Yes |
| Harris 2018 | Yes | Yes | Yes | Yes | Yes | - | Yes | Yes | Yes | Yes | Yes | Yes | Yes |
| Kullberg 2018 * | - | - | - | - | - | - | - | - | - | - | - | - | - |
| Ma 2006 | Yes | Yes | Yes | Yes | - | - | Yes | Yes | Yes | Yes | Yes | Yes | Yes |
| Marjerrison 2022a * | Yes | - | - | Yes | Yes | Yes | - | - | Yes | - | - | - | Yes |
| Marjerrison 2022b * | - | Yes | Yes | - | - | - | Yes | Yes | - | - | Yes | Yes | - |
| Sritharan 2022 | Yes | Yes | Yes | Yes | Yes | Yes | Yes | Yes | Yes | Yes | Yes | Yes | Yes |
| Petersen 2018a | Yes | Yes | - | Yes | Yes | Yes | Yes | Yes | Yes | Yes | Yes | Yes | Yes |
| Pukkala 2014 * | Yes^†^ | - | - | Yes^†^ | Yes^†^ | Yes^‡^ | Yes^†^ | - | - | - | - | - | Yes^†^ |
| Webber 2021 | Yes | - | - | Yes | Yes | - | Yes | - | Yes | - | - | Yes | Yes |
| Zeig-Owens 2011 | - | Yes | Yes | - | - | - | - | Yes | - | Yes | - | - | - |
| **No. of studies** | 14 | 12 | 10 | 14 | 12 | 7 | 14 | 11 | 12 | 10 | 11 | 10 | 13 |

* Some results may have been excluded from analyses of a given cancer site due to study population overlap with another study.

† Includes results for Finland and Iceland only.

‡ Includes results for Finland only.

Abbreviations: Meso, mesothelioma; NHL non-Hodgkin lymphoma

Supplemental Table C. Studies included in Table 2 meta-analyses for each cancer site (mortality outcomes)

| Study | Cancer site | | | | | | | | | | | | |
| --- | --- | --- | --- | --- | --- | --- | --- | --- | --- | --- | --- | --- | --- |
|  | All-cancer | Lung | Meso. | Prostate | Testis | Melanoma | NHL | Colon | Brain | Thyroid | Stomach | Bladder | Kidney |
| Ahn and Jeong 2015 | Yes | Yes | - | - | - | - | - | - | - | - | Yes | - | - |
| Amadeo 2015 | Yes | Yes | - | Yes | - | - | - | Yes | - | - | Yes | Yes | Yes |
| Aronson 1994 | Yes | Yes | - | Yes | Yes | Yes | Yes | Yes | Yes | Yes | Yes | Yes | Yes |
| Baris 2001 * | - | - | - | - | - | - | - | - | - | - | - | - | - |
| Bates 2001 | - | Yes | - | - | - | Yes | - | Yes | Yes | - | Yes | Yes | - |
| Beaumont 1991 * | - | - | - | - | - | - | - | - | - | - | - | - | - |
| Daniels 2014 * | - | - | - | - | - | - | - | Yes | - | - | - | - | - |
| Demers 1992 | Yes | Yes | - | Yes | - | - | Yes | - | Yes | - | Yes | Yes | Yes |
| Deschamps 1995 | Yes | - | - | - | - | - | - | - | - | - | - | - | - |
| Eliopulos 1984 | Yes | - | - | - | - | - | - | - | - | - | - | - | - |
| Glass 2016 | Yes | - | - | - | - | - | - | - | - | - | - | - | - |
| Guidotti 1993 | Yes | Yes | - | Yes | - | - | - | - | Yes | - | Yes | Yes | Yes |
| Hansen 1990 * | - | Yes | - | - | - | - | - | - | - | - | - | - | - |
| Heyer 1990 * | - | - | - | - | - | - | - | - | - | - | - | - | - |
| Ma 2005 | Yes | Yes | - | Yes | - | - | Yes | Yes | Yes | Yes | Yes | Yes | - |
| Marjerrison 2022b | Yes | Yes | Yes | Yes | Yes | Yes | Yes | Yes | Yes | Yes | Yes | - | Yes |
| Mastromatteo 1959 | Yes | - | - | - | - | - | - | - | - | - | - | - | - |
| Musk 1978 | Yes | - | - | - | - | - | - | - | Yes | - | - | - | - |
| Petersen 2018b | Yes | - | - | Yes | - | - | - | - | - | - | Yes | - | - |
| Pinkerton 2020 | Yes | Yes | Yes | Yes | Yes | - | Yes | - | Yes | Yes | Yes | Yes | Yes |
| Tornling 1994 | Yes | Yes | - | Yes | - | - | - | Yes | Yes | - | Yes | - | Yes |
| Vena and Fiedler 1987 | Yes | - | - | Yes | - | - | - | Yes | Yes | - | Yes | Yes | Yes |
| Zhao 2020 | Yes | Yes | Yes | Yes | - | Yes | - | Yes | Yes | - | Yes | Yes | Yes |
| **No. of studies** | 18 | 12 | 3 | 11 | 3 | 4 | 5 | 9 | 11 | 4 | 13 | 9 | 9 |

* Some results may have been excluded from analyses of a given cancer site due to study population overlap with another study.

Abbreviations: Meso, mesothelioma; NHL non-Hodgkin lymphoma

Supplemental Figure 2. Funnel plots for studies included in Table 2 analyses for overall and site-specific cancer incidence and overall cancer mortality


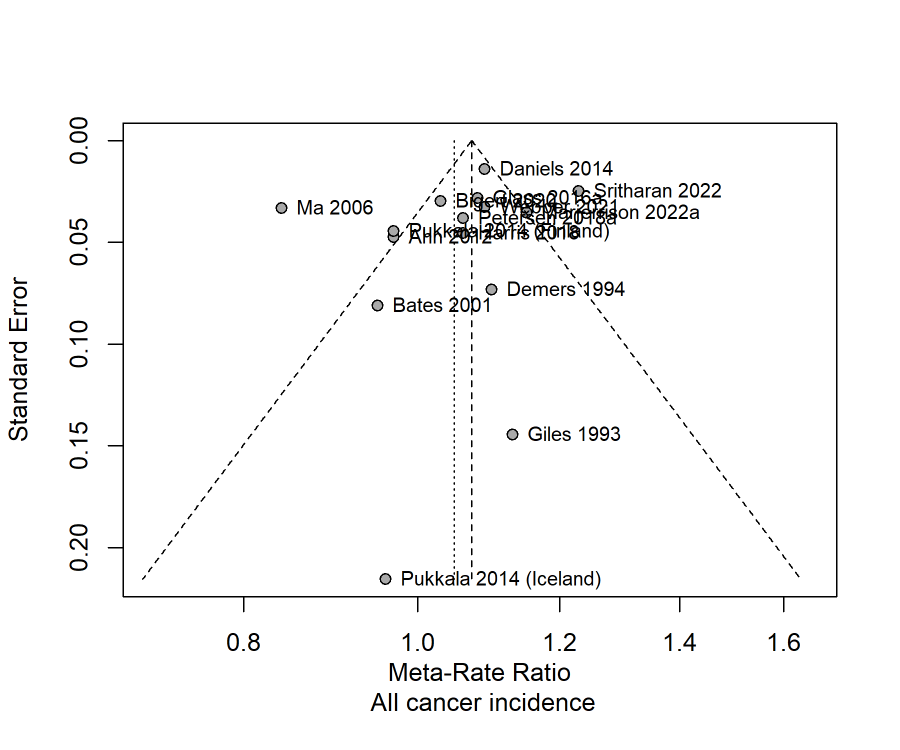

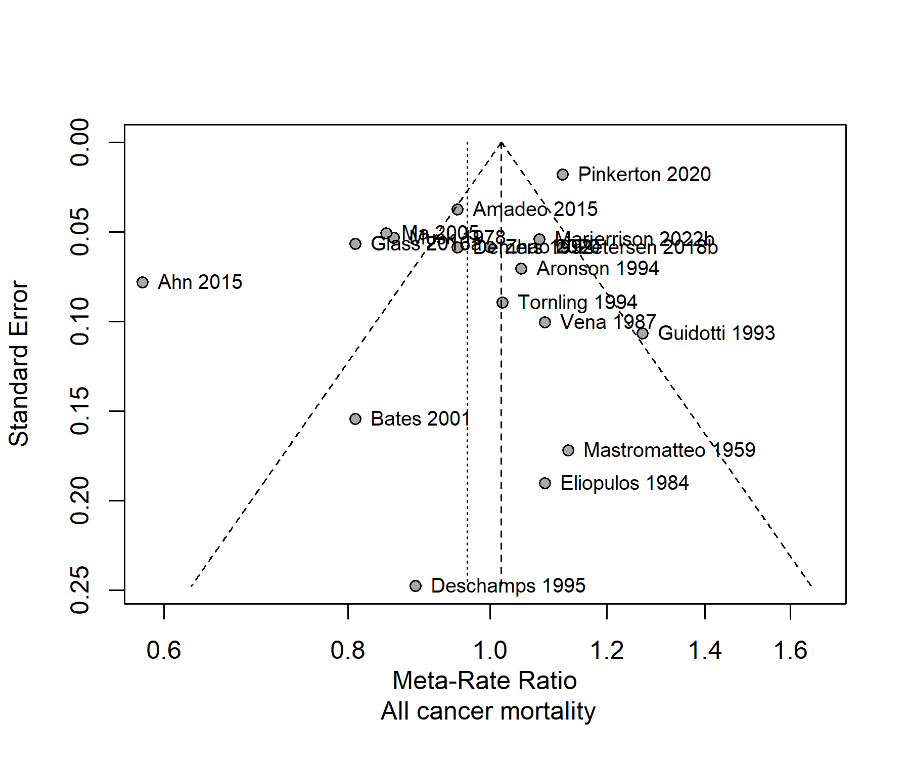

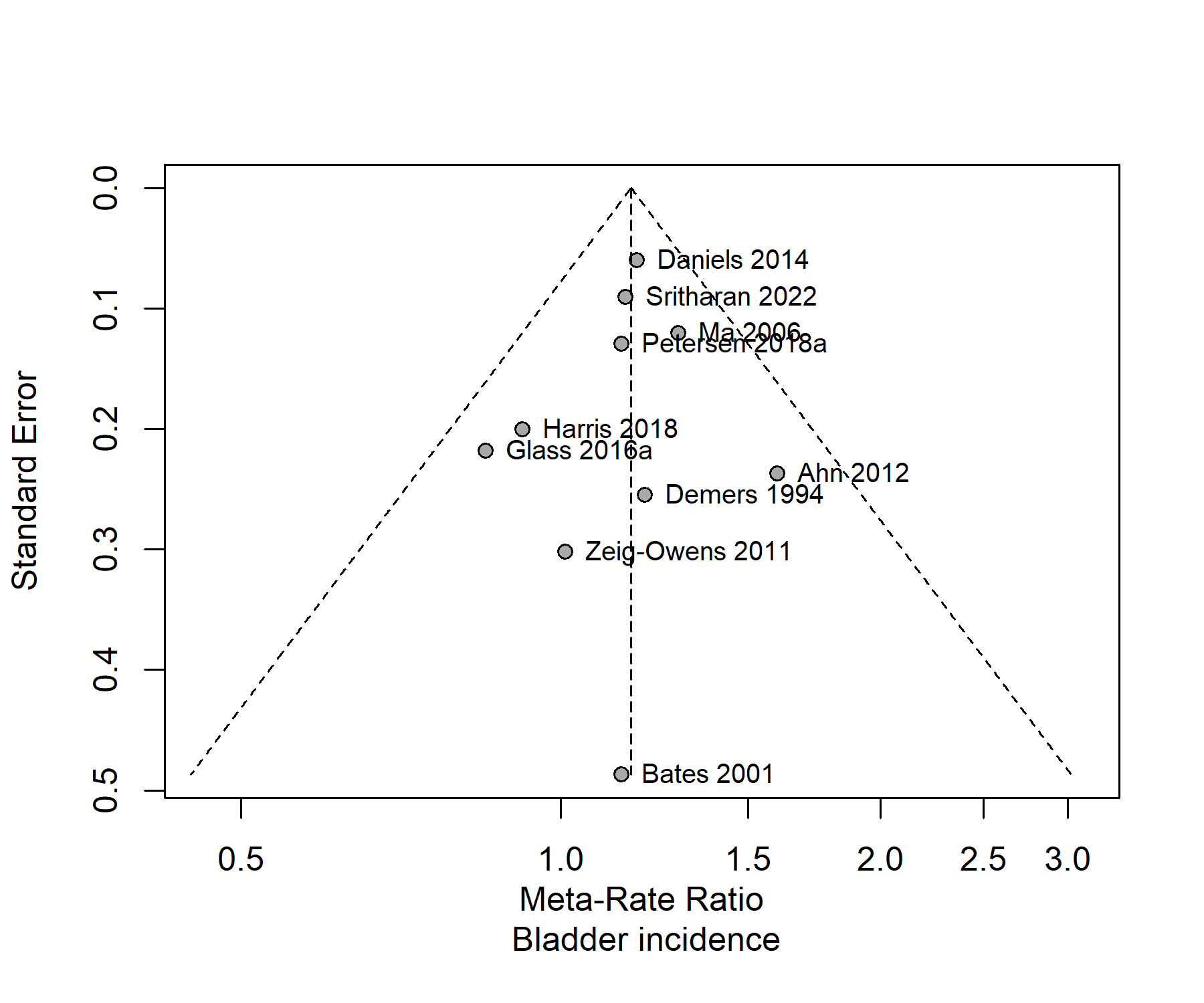

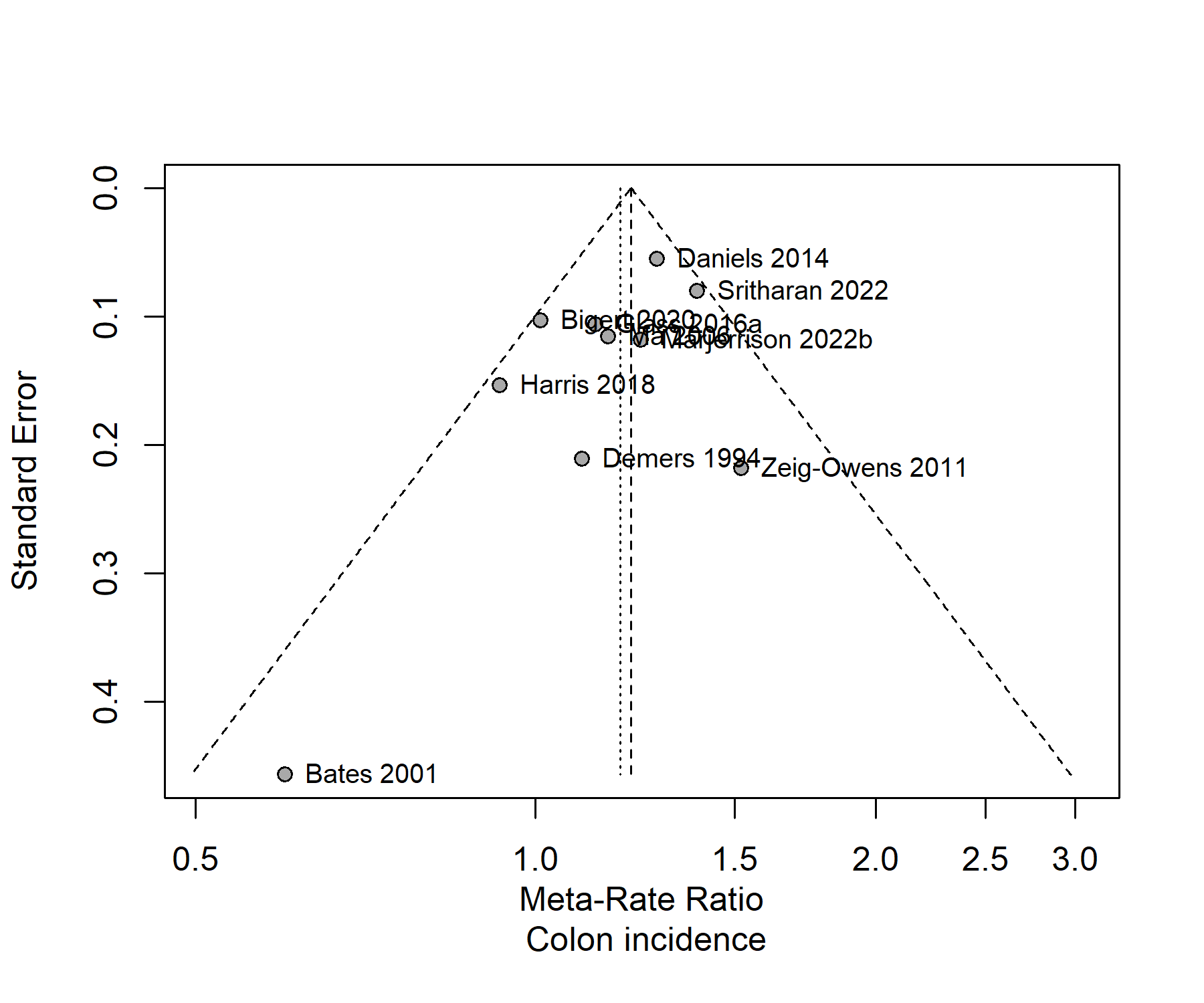

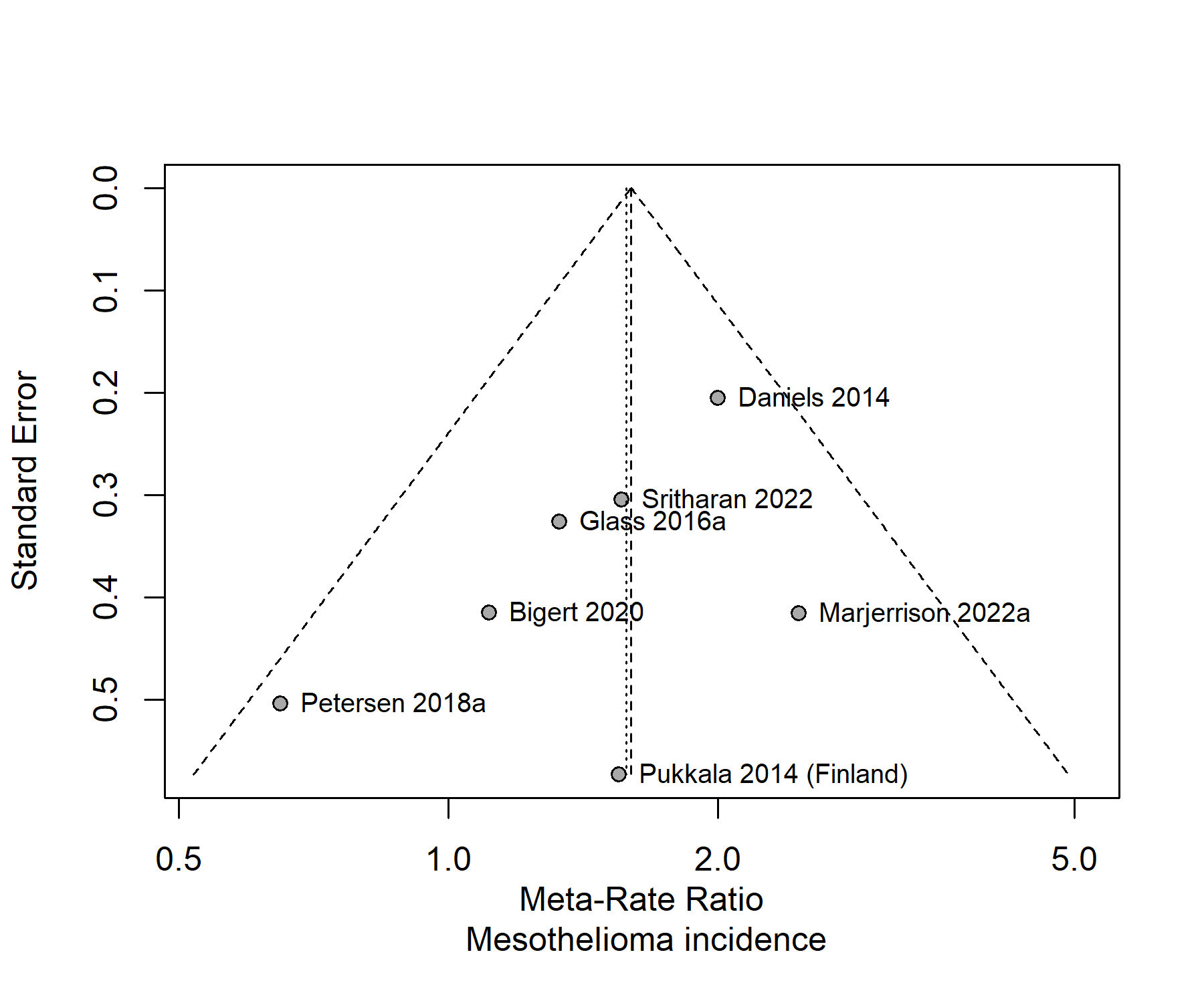

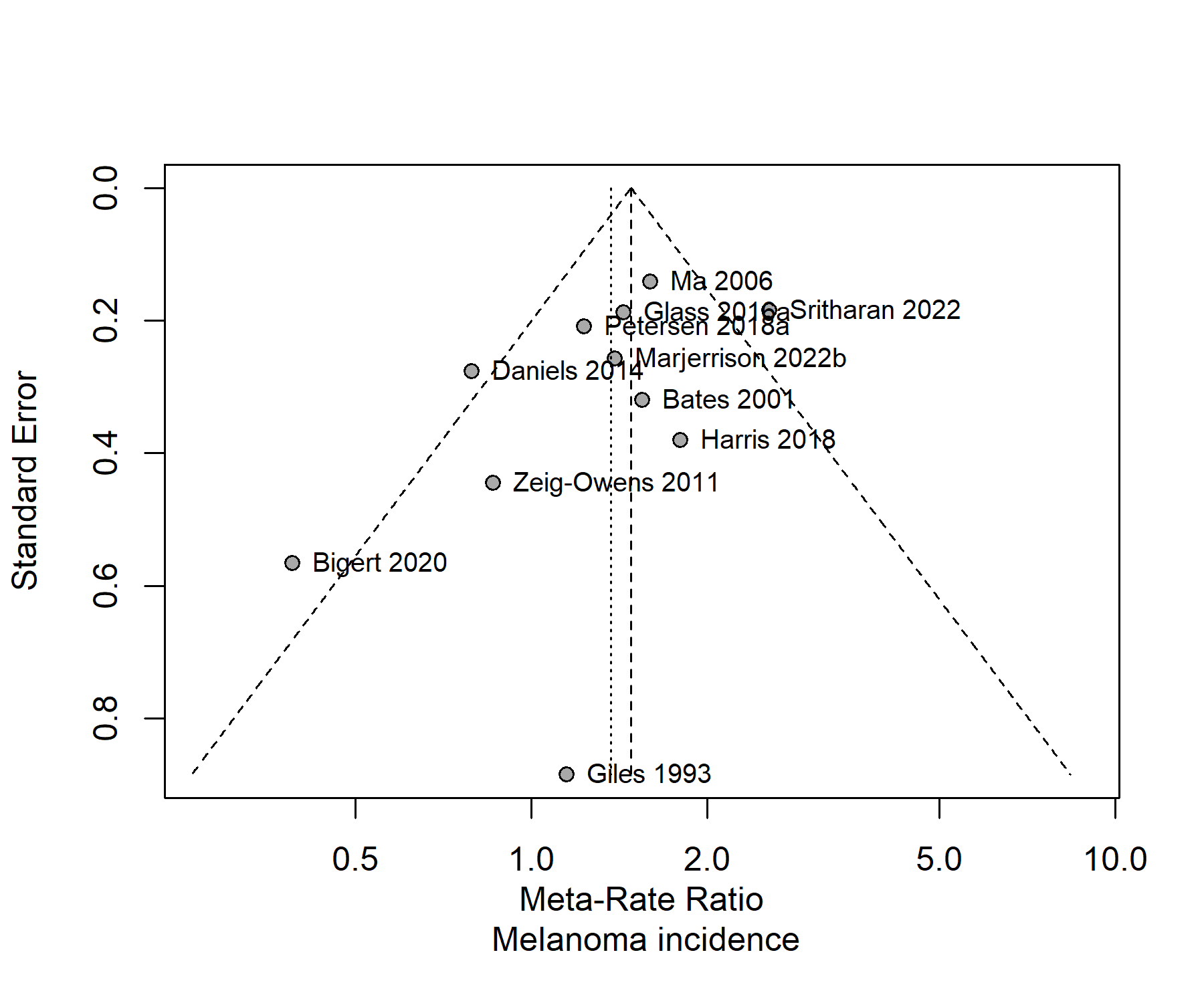


Supplemental Figure 3. Forest plots of individual study results and meta-rate ratios for select cancers among male career firefighters compared to a general, uniformed service, or working population referent †


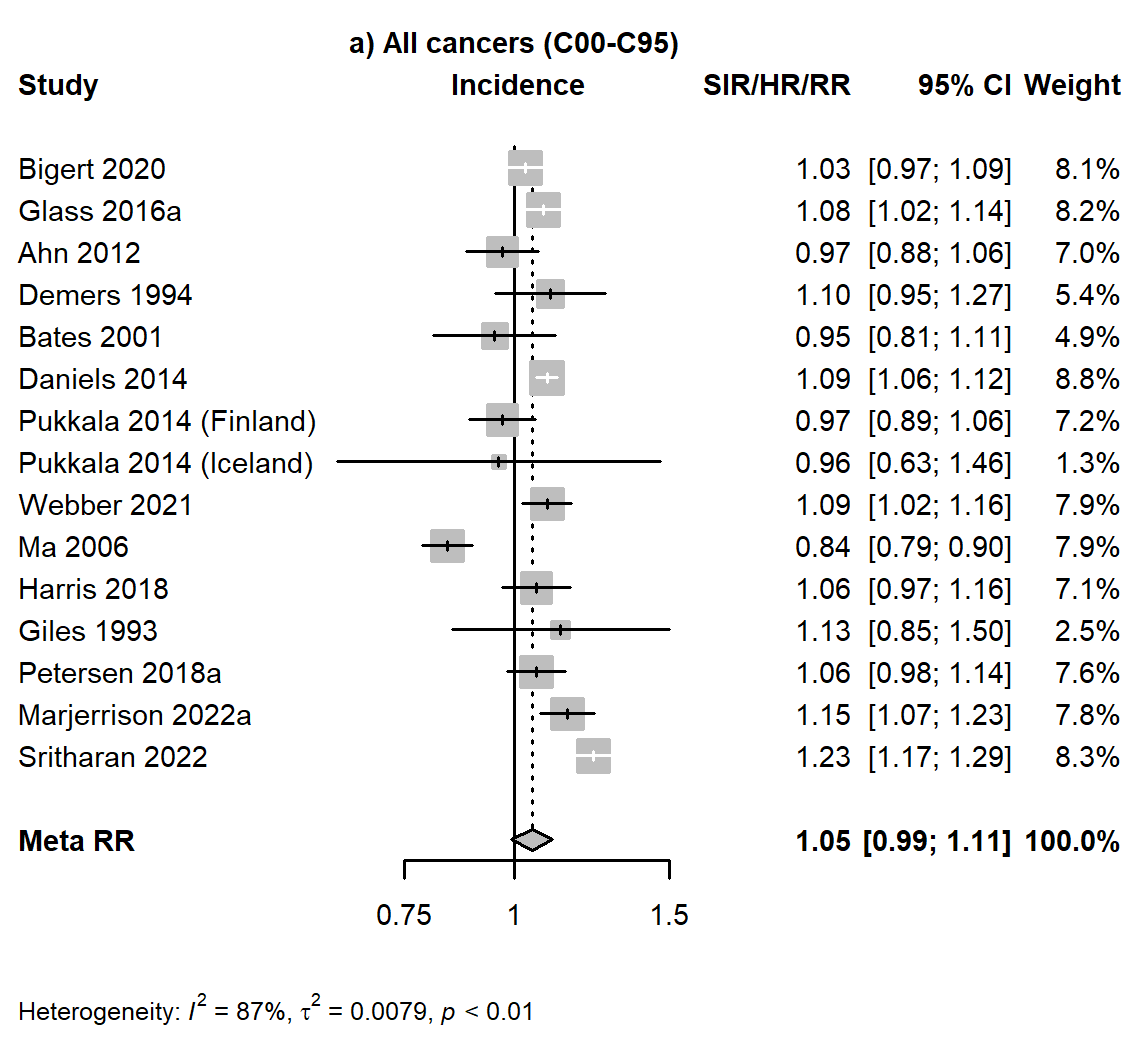

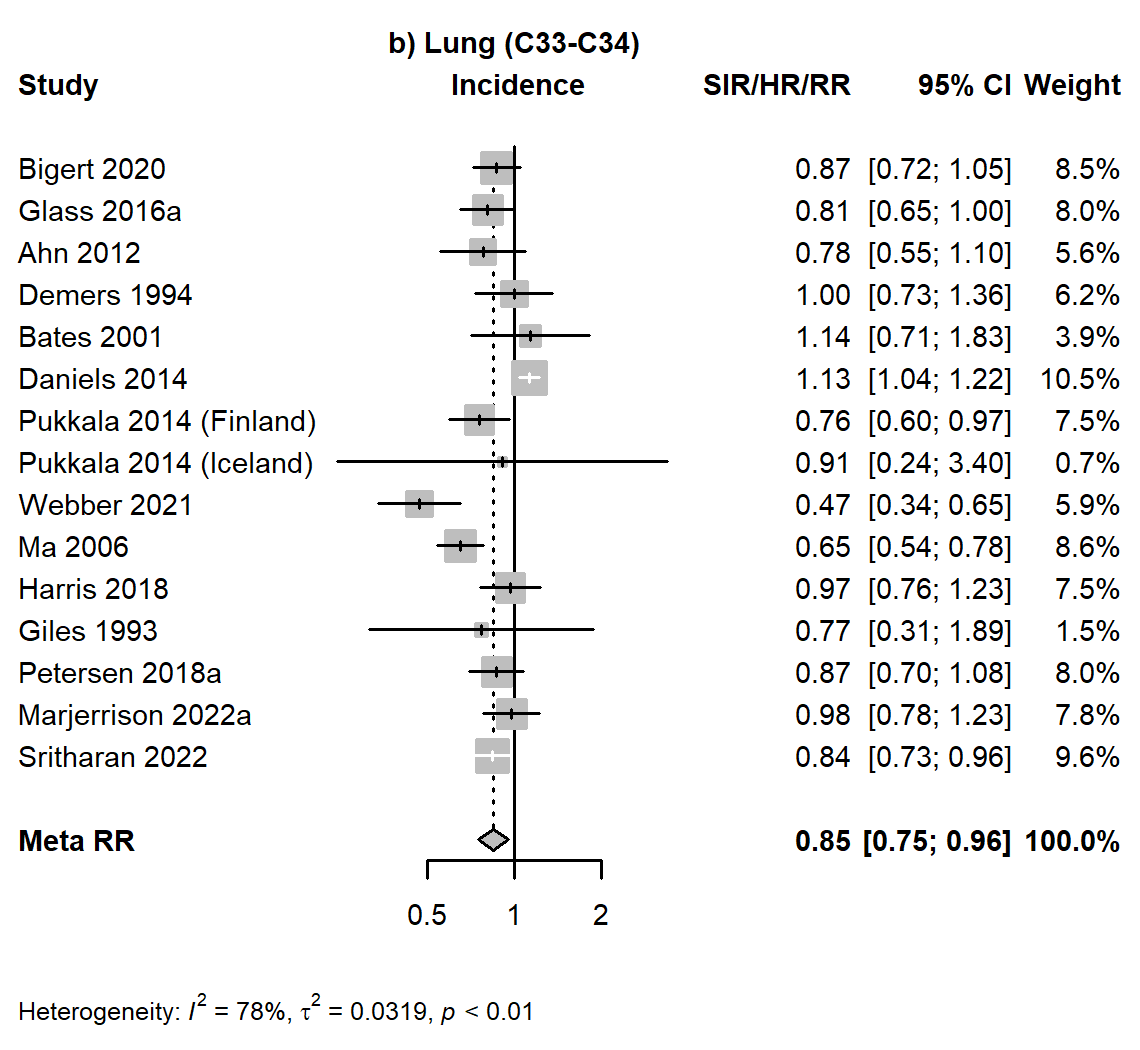

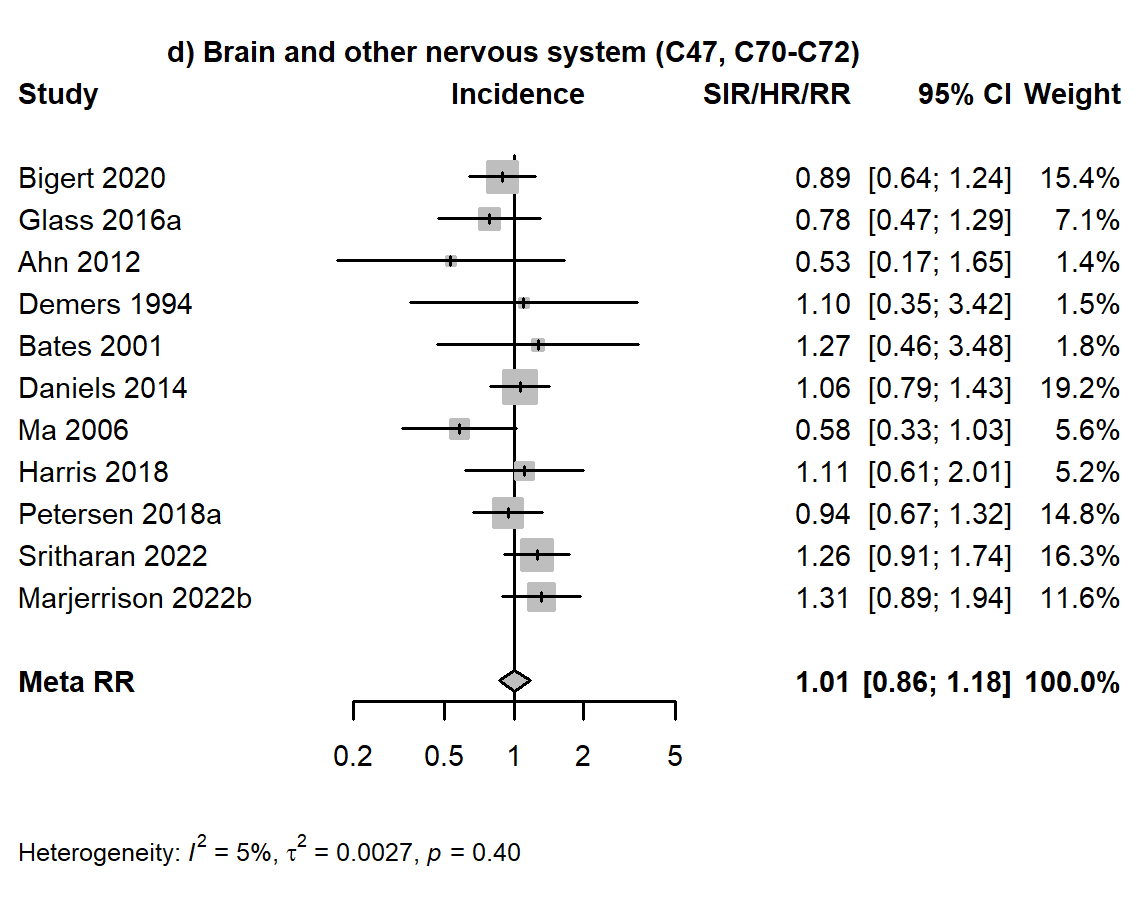

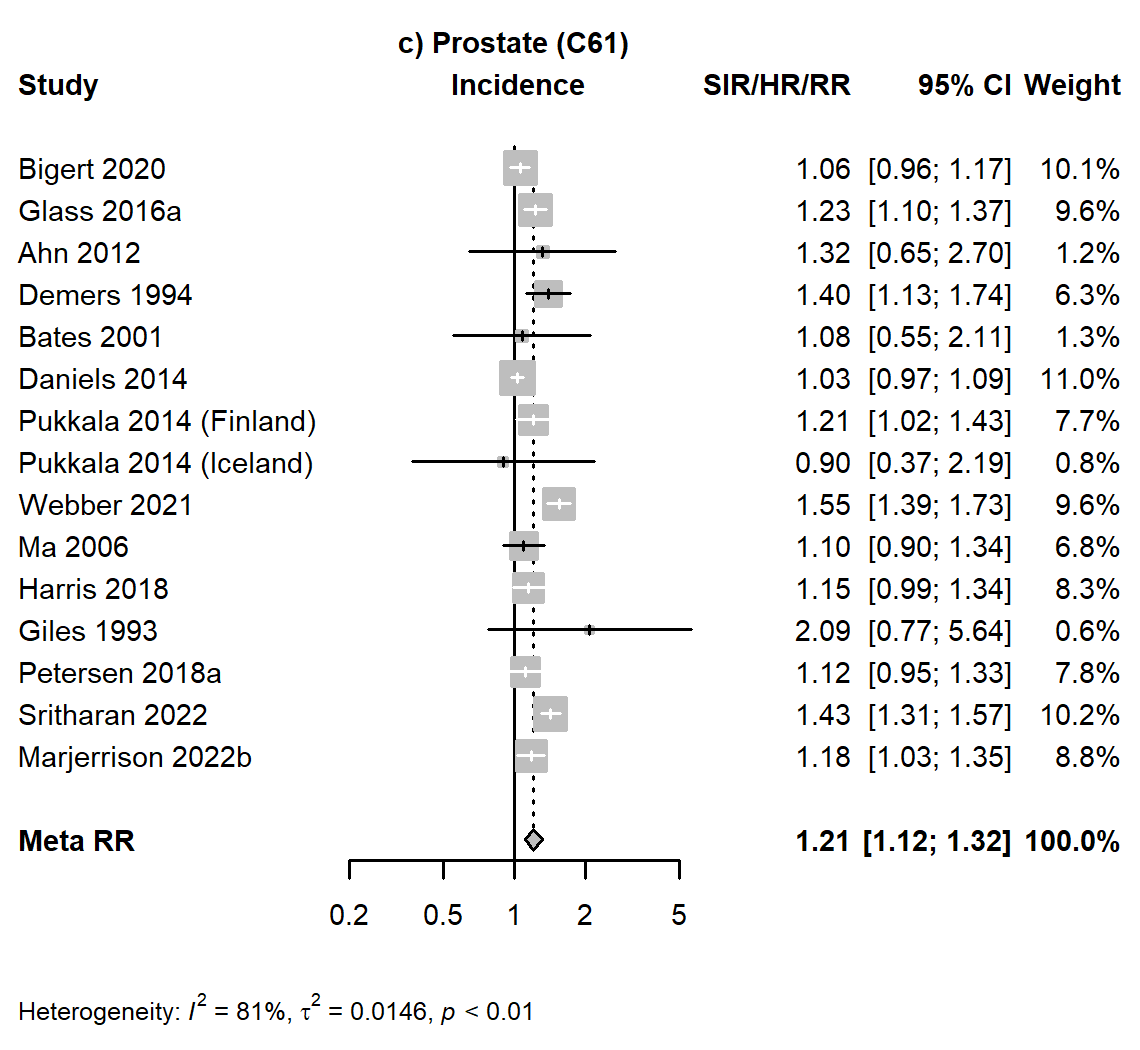

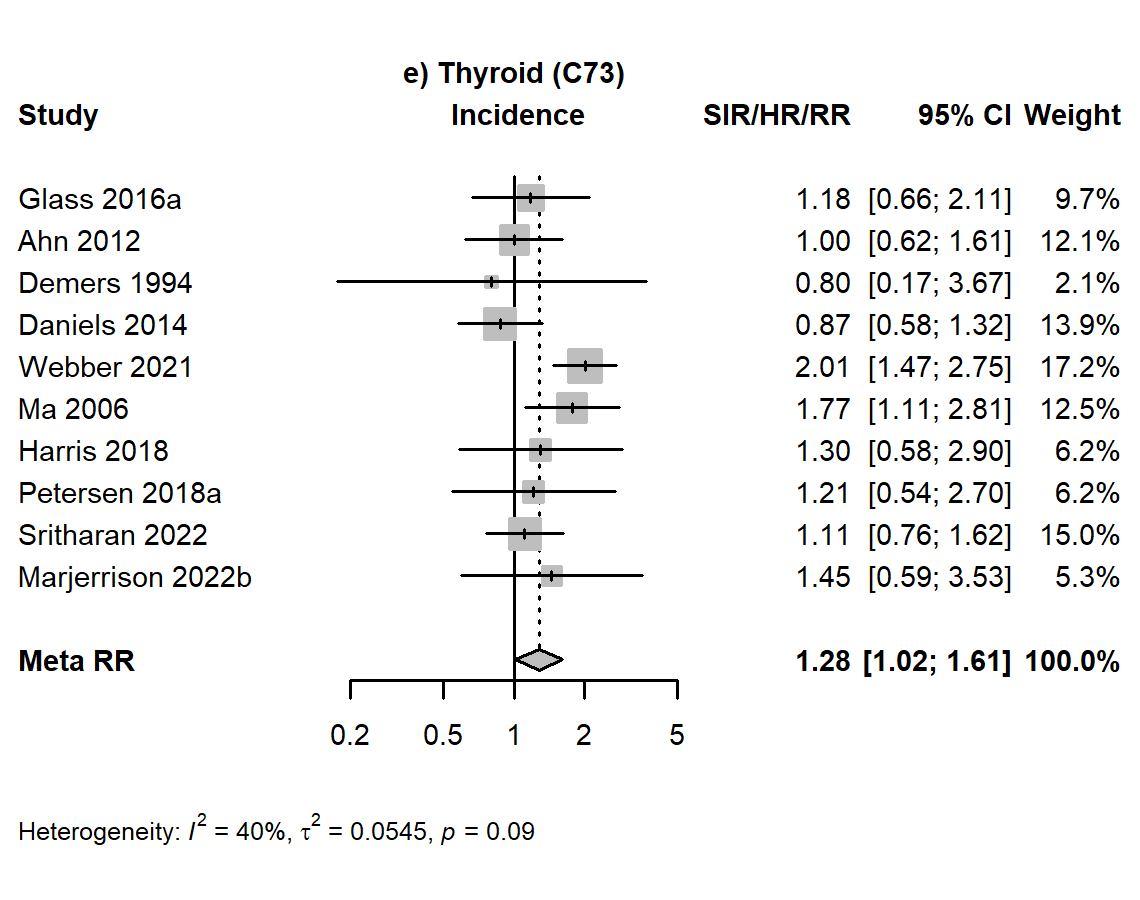

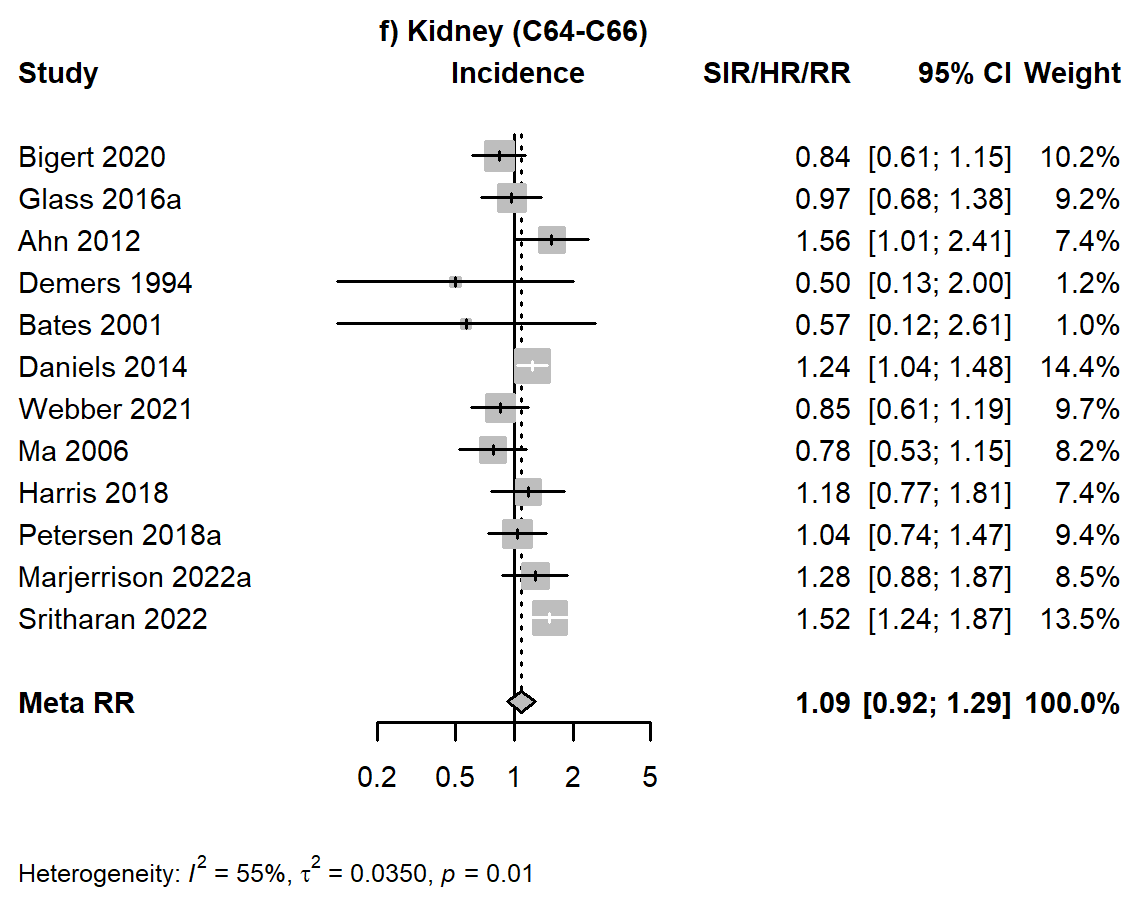


† Random-effects models were used with the restricted maximum-likelihood estimator. Knapp-Hartung adjustments and an ad hoc variance correction were used to calculate confidence intervals. Individual study variance is based on reported confidence interval bounds and may be slightly overestimated for estimates with few cases.

Supplemental Table D. Meta-Rate Ratios (mRR) for select cancers by duration of employment among male career firefighters compared to the general population

| Outcome | Studies  (n) | Obs * | Employment duration  mRR (95% CI) † | | | I^2^  (%) | Q  *p*-value | Subgroup difference  *p*-value ‡ |
| --- | --- | --- | --- | --- | --- | --- | --- | --- |
|  |  |  | **<10 yrs** | **10-20 yrs** | **>20 yrs** |  |  |  |
| **Incidence** § |  |  |  |  |  |  |  |  |
| All cancers (C00-C95) | 8 | 23 | 1.06 (0.94-1.18) | 1.03 (0.92-1.15) | 1.06 (0.99-1.13) | 28 | 0.11 | 0.85 |
| Stomach (C16) | 4 | 13 | 1.05 (0.60-1.86) | 0.97 (0.70-1.35) | 1.08 (0.74-1.57) | 0 | 0.93 | 0.82 |
| Colon (C18) | 3 | 10 | 0.92 (0.19-4.37) | 0.95 (0.28-3.26) | 1.18 (0.84-1.65) | 0 | 0.99 | 0.67 |
| Lung (C33-C34) | 7 | 22 | 1.03 (0.76-1.40) | 0.97 (0.73-1.31) | 0.85 (0.73-1.00) | 0 | 0.58 | 0.34 |
| Melanoma (C43) | 6 | 18 | 1.39 (0.92-2.10) | 1.44 (1.04-1.99) | 1.36 (1.16-1.59) | 0 | 0.98 | 0.92 |
| Mesothelioma (C45) | 3 | 9 | 6.38 (0.53-77.20) | 2.01 (0.37-11.05) | 1.44 (0.63-3.28) | 19 | 0.27 | 0.15 |
| Prostate (C61) | 6 | 18 | 1.19 (0.88-1.60) | 1.02 (0.76-1.37) | 1.19 (1.04-1.37) | 0 | 0.59 | 0.36 |
| Testis (C62) | 3 | 8 | 1.65 (0.64-4.27) | 1.58 (0.02->100) | 1.42 (0.44-4.62) | 0 | 0.45 | 0.91 |
| Kidney (C64­-C66) | 3 | 9 | 1.30 (0.27-6.18) | 1.33 (0.51-3.48) | 1.20 (0.62-2.32) | 0 | 0.84 | 0.92 |
| Bladder (C67-C68) | 4 | 11 | 1.34 (0.78-2.28) | 1.71 (0.63-4.68) | 0.88 (0.55-1.44) | 8 | 0.37 | 0.09 |
| Brain and nervous (C47, C70-C72) | 4 | 9 | 0.84 (0.08-8.44) | 0.65 (0.0->100) | 1.32 (0.44-3.99) | 0 | 0.56 | 0.53 |
| Non-Hodgkin lymphoma (C82-C85) | 6 | 18 | 0.90 (0.54-1.49) | 1.20 (0.82-1.75) | 1.07 (0.85-1.45) | 0 | 0.95 | 0.62 |
| **Mortality** |  |  |  |  |  |  |  |  |
| All cancers (C00-C97) | 7 | 19 | 0.96 (0.54-1.69) | 0.85 (0.50-1.44) | 1.00 (0.78-1.28) | 79 | <0.01 | 0.62 |
| Stomach (C16) | 3 | 9 | 0.86 (0.02-34.49) | 0.73 (0.02-25.01) | 1.04 (0.61-1.78) | 43 | 0.08 | 0.45 |
| Lung (C33-C34) | 5 | 15 | 0.94 (0.52-1.69) | 0.92 (0.43-1.99) | 1.01 (0.68-1.50) | 18 | 0.25 | 0.46 |
| Prostate (C61) | 3 | 9 | 1.46 (0.89-2.42) | 1.08 (0.64-1.83) | 1.08 (0.93-1.26) | 0 | 0.87 | 0.42 |
| Brain and nervous (C47, C70-C72) | 5 | 14 | 1.49 (0.31-7.11) | 2.10 (0.50-8.81) | 2.23 (1.16-4.26) | 51 | 0.01 | 0.42 |

* Total number of included effect-estimates across all duration of employment categories

† Estimates in each duration of employment subgroup using random effects meta-analysis model

‡ Omnibus type 3 tests of fixed effects subgroup differences in categorical regression model

§ Outcomes with fewer than 3 available studies were not meta-analyzed

Abbreviations: mRR, meta-rate ratio; CI, confidence interval; Obs, observations; yrs, years.

Supplemental Table E. Meta-analysis of duration of employment specified as a continuous variable in meta-regression

| Outcome | Studies (n) | Obs. | Response * | | | Δ tau †  (%) ‡ | Heterogeneity  p-value § |
| --- | --- | --- | --- | --- | --- | --- | --- |
|  |  |  | **Intercept** | **Slope** | **p-value** |  |  |
| **Incidence** |  |  |  |  |  |  |  |
| All cancers (C00-C95) | 8 | 26 | 0.0351 | 0.0004 | 0.8003 | 0.0 | 0.0229 |
| Stomach (C16) | 4 | 13 | -0.0260 | 0.0023 | 0.7682 | 0.0 | 0.8973 |
| Colon (C18) | 3 | 10 | -0.1819 | 0.0109 | 0.3603 | 0.0 | 0.9975 |
| Lung (C33-C34) | 7 | 23 | 0.0259 | -0.006 | 0.2131 | -11.8 | 0.5753 |
| Melanoma (C43) | 6 | 19 | 0.3697 | -0.0021 | 0.6556 | 0.0 | 0.9830 |
| Mesothelioma (C45) | 3 | 9 | 1.7253 | -0.0398 | 0.1139 | -37.9 | 0.5327 |
| Prostate (C61) | 6 | 19 | 0.0637 | 0.0034 | 0.3257 | 28.8 | 0.6257 |
| Testis (C62) | 3 | 9 | 0.5564 | -0.0091 | 0.4606 | -28.7 | 0.2823 |
| Kidney (C64­-C66) | 3 | 9 | 0.2338 | -0.0004 | 0.9729 | 0.0 | 0.7603 |
| Bladder (C67-C68) | 4 | 12 | 0.4845 | -0.0174 | 0.0597 | -87.3 | 0.7504 |
| Brain (C47, C70-C72) | 4 | 9 | -0.3378 | 0.0180 | 0.3759 | -44.1 | 0.6152 |
| NHL (C82-C85) | 6 | 19 | 0.1266 | -0.0021 | 0.7969 | 0.0 | 0.9472 |
| **Mortality** |  |  |  |  |  |  |  |
| All cancers (C00-C97) | 7 | 22 | -0.2441 | 0.0077 | 0.1460 | -7.9 | <0.0001 |
| Stomach (C16) | 3 | 10 | -0.3838 | 0.0137 | 0.1226 | -42.8 | 0.2963 |
| Lung (C33­-C34) | 5 | 19 | -0.0749 | 0.0022 | 0.4803 | 0.0 | 0.3690 |
| Prostate (C61) | 3 | 11 | 0.3777 | -0.0088 | 0.1825 | 93.3 | 0.8795 |
| Brain (C47, C70-C72) | 5 | 16 | 0.4353 | 0.0126 | 0.2051 | -5.7 | 0.0209 |

* Variables from a model of the form ln(mRR) = α + βd, where α is the model intercept, d is the employment duration in years, and β is the slope parameter of the exposure-response relationship.

† Omnibus Type 3 test of fixed effect.

‡ Relative change (%) in the residual heterogeneity from adding the exposure covariate to the model.

§ Obtained from a Q test.

Abbreviations: mRR, meta-rate ratio; obs, observations

Supplemental Methods Information

*Literature search and exclusion criteria*

A systematic search was conducted of the PubMed, Web of Science, and Embase literature databases to identify epidemiological studies of the association between occupational exposure as a firefighter and the occurrence of cancer incidence or mortality in humans. Occupational exposure as a firefighter was defined as any exposure to the occupation regardless of employment type (e.g., career, part-time, seasonal, volunteer) or activities performed (e.g., wildland or structural firefighting, disaster response). The exact search terms, results, and a flowchart illustrating the number of excluded studies is available in supplemental material (Suppl. Table A, Figure 1). Briefly, our search used English ‘MeSH’ terms and keywords related to firefighting and cancer, but articles written in any language were translated and considered for inclusion. Only studies published in peer-reviewed scientific journals were eligible. Searches were supplemented by authors’ reviews of reference lists for cited studies, including the previous monograph. The search included articles published until 13 June 2022 and led to the identification of a total of 644 studies involving firefighters that were considered for inclusion in the meta-analysis.

The abstracts of all 644 studies were reviewed by two co-authors (N.D. and M.S-B.) to apply exclusion criteria. Studies were excluded at this stage (Suppl. Fig 1) if they did not report the occurrence of cancer as an outcome (n = 444), used a cross-sectional or ecological (n = 3), case report (n = 13), or meta-analysis (n = 7) study design, were conference abstracts or duplicates of an existing study (n = 15), presented no primary estimates of association between firefighting and cancer (n = 41), were letters or commentaries (n = 21), or if they were general occupational surveillance studies that did not investigate cancer in firefighters *a priori* (n = 37). The exclusion of such occupational surveillance studies was done to reduce the influence of publication bias on the meta-analysis, given that these studies could not be found systematically via abstract-only literature searching and were generally only identified if positive findings for firefighters were highlighted.

The remaining 63 studies received detailed full-text review. For studies describing previous analyses of populations that were subsequently updated with additional follow-up, those that did not report the most recent results for a given cancer site or analysis type were excluded. One study was excluded for having an analytic error that produced invalid results [personal communication]. Only one population-based case-control study met inclusion criteria, but it was excluded from meta-analyses with cohort studies to reduce heterogeneity of estimated effects due to marked differences in study design. Results from this study were instead synthesized qualitatively with meta-analysis results.

*Bias assessment*

A bias assessment tool was developed to evaluate the potential influence of six biases determined through the Working Group’s judgement to be most relevant to epidemiological studies of occupational exposure as a firefighter and cancer. This approach was preferred over the use of standardized checklists for evaluating study quality, which can induce arbitrary thresholds based on numeric ratings and diminish the relative strengths of available studies on a given topic. The chosen bias domains were misclassification of exposure, misclassification of outcome, healthy worker hire and survivor bias, confounding by lifestyle factors (e.g., tobacco or alcohol consumption, sun exposure) or occupational exposures outside of firefighting, medical surveillance bias, and selection bias. Six co-authors (R.D., L.B-F., J.G., J.H, D.G., D.K.) independently evaluated each study according to their level of concern (‘major’, ‘moderate’, or ‘minor’) about the influence of each source of bias on the magnitude and direction of effect estimates reported for each cancer site. Two additional co-authors (L.S., M.S-B.) reviewed conflicting assessments and each conflict was discussed by all eight authors involved to reach consensus on the final assessments. Studies with a ‘major’ level of concern for one or more bias domains were excluded in sensitivity analyses (described below) to determine the impact of results from these studies on the meta-effect estimates.

Studies that were based exclusively on populations of decedents or cancer cases without enumeration and follow-up of the population from which the cases developed, such as proportionate mortality ratio studies and studies nested within a cancer registry, were assessed as having ‘major’ concern for both exposure misclassification and selection bias. This was due to the incomplete ascertainment of occupational information in these data sources and the disproportionate selection of individuals with cancer into the overall study population. As a result of these influential sources of bias, 11 ‘event-only’ studies of this type were excluded from further assessment in the meta-analysis.

*Data analysis*

The objective of the analysis was to meta-analyze the association between ever-employment and duration of employment as a firefighter and cancer incidence and mortality. Information for studies selected from the bias assessment exercise was extracted into a dataset by three co-authors (N.D., R.W., A.M.-F.) using a combination of manual data entry and automated software. Given that several studies were conducted in the same country, results determined to have a high potential for overlapping study populations were considered for exclusion to reduce underestimation of the standard error of meta-effect estimates. Where substantial overlap was considered possible for a given analysis or outcome, results from studies with a shorter length of follow-up or fewer cases were excluded (Suppl. Table B & C). Studies also varied according to the types of analyses conducted, with several reporting analyses of firefighters with different employment types (e.g., full-time versus part-time/volunteer), external comparison populations (e.g., general population, police, military, other workers), exposure metrics (e.g., cumulative fire responses), and distributions of males and females. To reduce the heterogeneity of estimates included in the meta-analysis with respect to some of these important characteristics, results based exclusively on females or part-time/volunteer firefighters were excluded from analyses. Results for female firefighters were too few for stratified meta-analysis. Results from internal comparison analyses according to metrics of firefighting exposure were also excluded as few studies conducted such analyses, and the type of reported exposure metrics varied.

There were 13 cancer sites chosen for analysis identified *a priori* from the studies in the systematic literature review and the results from previous meta-analyses. Of the chosen sites, results were grouped from available studies according to the 10th revision of the International Classification of Diseases (ICD) and were intended to be focused on the target organ to the greatest extent possible. Exceptions were made for lung and bladder cancer, which included trachea (C33) and other urinary cancers (C68) respectively, since several studies used these expanded case definitions and inclusion of these additional cancer sites was not expected to meaningfully affect risk interpretations for the target organ. Cancer mortality and incidence outcomes were analyzed separately. Combining these outcomes for highly fatal cancers (e.g., lung cancer, mesothelioma) did not meaningfully change results (not shown). The specific studies included in the meta-analyses for each cancer site are listed in Suppl. Table B and C.

The meta-effect estimates, referred to henceforth as meta-rate ratios (mRR), were estimated with inverse-variance weighted random-effects models and the natural logarithm of the reported study effect estimates. Estimates of within-study variance were obtained from the reported 95% confidence interval (CI) bounds and therefore may be slightly overestimated for results based on few cases. Missing CIs were calculated from observed and expected cases using exact methods. The between-study variance (τ^2^) was estimated using restricted maximum-likelihood (REML) methods. Residual heterogeneity (i.e., the variability of effect-estimates attributable to sources other than random sampling error) was described by the I^2^ statistic and Q test *p*-value. The Hartung-Knapp-Sidik-Jonkman (HKSJ) method was used to calculate 95% CIs unless the interval was narrower than that using standard random-effects methods. Funnel plots were examined for evidence of reporting bias and are shown in Suppl. Figure 2. All analyses were conducted using the ‘meta’ package in R Statistical Software version 4.1.2.

The main analysis consisted of results for the association between ever-employment as a firefighter and cancer using any population as the referent. If results using more than one referent population were available in a given study, those using the general population were chosen as they were the most common. All studies in the main analysis were cohort studies following firefighters for cancer over time and reporting Standardized Incidence/Mortality Ratio (SIR, SMR), Incidence Rate Ratio (RR), or Hazard Ratio (HR) effect measures. A secondary analysis consisted of results for the association between duration of employment as a firefighter and cancer using a three-level mixed effect model in both categorical meta-analysis and meta-regression. Duration of employment was categorized into three groups (<10, 10-20, >20 years) with associations in each group aggregating reported estimates that fit entirely within these categories. The three-level model was used to account for correlation between multiple estimates within a single study. To better assess the slope of the trend between duration of employment and cancer and to include all estimates that spanned multiple duration categories, duration was also specified as a continuous variable in a meta-regression model using the midpoint of the reported categories and assuming a maximum of a 45-year career length for open-ended categories. Differences in duration subgroups were examined by inspection of forest plots, omnibus test, and inspection of plotted trend lines in meta-regression.

*Sensitivity analyses*

Given the diversity of study populations, referent groups, exposure definitions, lengths of follow-up, and methods of outcome ascertainment across studies included in the main analysis, we sought to elucidate sources of bias and heterogeneity in mRR estimates by using triangulation methods in sensitivity analyses. The effect of using different referent populations was explored by including results using uniformed service (e.g., police, military, other firefighters) or working populations as reference groups instead of the general population when both were available. Stratified analyses were also done with restriction to studies using a general population referent only and to studies using uniformed service referent populations only. Restriction was also applied to only include studies with an average age at end of follow-up of ≥55 years or average follow-up length of >20 years (when age not reported) to evaluate the influence of excluding studies that primarily observed cancer occurrence during younger, lower-risk age windows. Studies assessed as having a ‘major’ level of concern for any of the six bias domains in the bias assessment exercise were also excluded separately to evaluate the potential impact of these sources of bias on results in the main analysis.
